# An end to the controversy over the microscopic detection and effects of pristine microplastics in fish organs

**DOI:** 10.1038/s41598-020-69062-3

**Published:** 2020-07-24

**Authors:** Carolina De Sales-Ribeiro, Yeray Brito-Casillas, Antonio Fernandez, María José Caballero

**Affiliations:** 1 Division of Veterinary Histology and Pathology, Institute for Animal Health and Food Safety (IUSA), Veterinary School, University of Las Palmas de Gran Canaria, 35413 Arucas, Spain; 2 Research Institute in Biomedical and Health Sciences (IUIBS), University of Las Palmas de Gran Canaria, 35016 Las Palmas de Gran Canaria, Spain

**Keywords:** Zoology, Environmental sciences

## Abstract

The aquatic environment and the associated fish assemblages are being exposed to an increasing amount of microplastics. Despite the high number of publications on the presence of microplastics in fish, little is known about their uptake, translocation and accumulation within fish organs. Experimental studies on the detection and effects of pristine microplastics in fish have shown controversial and ambiguous results, respectively. Here, we conducted two experiments to detect and assess the impacts of dietary exposure of *Danio rerio* to different types of pristine microplastics. Our results show that *D. rerio* recognizes plastic particles as inedible materials but ingests them when mixed with food or fish oil. Accidental ingestion occurs in fish exposed to relatively small (1–5 µm) microplastic particles without associated food or fish oil. Additionally, *D. rerio* effectively eliminated pristine microplastics 24 h after ingestion; however, retention time was associated with increasing particle size and the intake of additional meals. Clinical signs, such as anorexia and lethargy, are present in fish fed relatively large microplastics (120–220 µm). The ingestion of microplastics does not induce any histopathological changes. To the best of our knowledge, we are able, for the first time, to fully demonstrate the uptake and translocation of plastic microbeads using confocal microscopy. Our results question the findings of previous studies on the detection and effects of pristine microplastics in fish and state that inaccurate interpretations of the histological findings regarding microplastics in fish organs is a prevalent flaw in the current scientific literature.

## Introduction

The ever-growing production of plastics^1,2^ and their relatively short lifespan^3^, combined with indiscriminate waste-disposal practices and accidental releases^1^, have led to the accumulation of plastics in aquatic environments worldwide^4–9^. This situation is especially worrisome due to their long degradation time^2^ and potential to be ingested by aquatic organisms^3^.

In water, plastics undergo weathering^10^ through photolytic, mechanical and biological degradation^11,12^. Under these circumstances, larger plastics degrade into smaller fragments^12^, i.e., microplastics (MPs) (< 5 mm)^13^. Another source of MPs in aquatic environments is micro-sized particles intentionally manufactured for use in domestic products (e.g., cosmetics and clothing) and industrial products (media blasting and industrial feedstock)^11,12^, which are directly introduced into the environment by human activity^10^.

MPs can display a variety of shapes, sizes and colours^12^. In aquatic systems, the predominant shapes of MPs are fibres^14–18^, fragments^6,7,19,20^ and microbeads^21^. The small size and ubiquity of MPs^22^ makes them easily available to aquatic fauna, which are prone to ingest them by confusion with food, accidental ingestion or by transfer through the food chain^23,24^. Several studies have documented the ingestion of plastic and MP particles in aquatic species (invertebrates^10,25–28^, amphibians^29^, reptiles^30^, marine mammals^8,31^ and seabirds^32,33^).

In fish, the presence of MPs in the gastrointestinal tract (GIT) has been reported in several marine and freshwater species captured all around the world^34–38^. Overall, the average number of particles found in the GITs of fish ranged from 0 to 3 items/fish^34,36–44^. The low number of particles suggests that the potential for accumulation of MPs in the GITs of fish is close to zero and that the presence of MPs in the GITs is indicative of a recent ingestion^23^. However, to confirm this theory, further dietary experimental studies are needed. Knowing the retention times of MPs in fish GITs will help to determine the MP load to which fish are exposed in their lifetime^23^. An experimental study, in which *Carassius auratus* were fed a single intake of MPs (100–500 µm), showed that after 24 h, 90% of the plastic particles had been eliminated, with only 0 to 3 particles being excreted after 144 hours^45^. In another experiment with *Seriolella violacea,* MP (length, 1.2 ± 0.2 mm; diameter, 1.0 ± 0.1 mm) elimination took up to 7 weeks^46^. In *Sparus aurata* fed multiple meals with MPs, most particles were eliminated after a 30-day period of depuration^47^. In general, MPs appear to have a small long-term potential for accumulation, being unlikely to accumulate in the digestive tract^45,47,48^. However, larger particles seem to be retained for a longer time in the GIT. It is also important to bear in mind that the structure of the digestive tract varies among fish species^49^ and that additional intrinsic (species, age, and physiology) and extrinsic (habitat, food concentration/availability, and type of MPs) factors have to be taken into account when discussing retention rate patterns for MPs. All these variables highlight the necessity to carry out more studies in different species to better understand the potential for accumulation of MPs in fish.

Despite the apparent effective elimination, a study^24^ reported the uptake of MP particles (1–20 μm) by enterocytes in 3 out of 39 fish.

The translocation of MP particles to other tissues, such as the liver^47,50,51^ and muscle^37,47^, has been described. Avio et al. reported the translocation of plastic particles (200–600 μm) from the digestive tract to the liver in *Mugil cephalus*^50^. Likewise, Collard et al. documented the presence of two particles (39–90 μm) in the livers of *Engraulis encrasicolus*^51^, and Abbasi et al*.* detailed the presence of variably sized MP particles (up to 250 µm) in the livers of four fish species captured in the Persian Gulf^37^. Similarly, Jovanovic et al*.* observed the presence of < 1 particle (214–288 μm) in the liver of *S. aurata*^47^, carefully pointing to the possibility of cross-contamination. The alleged translocation of such large particles is difficult to explain with the current knowledge on translocation pathways for MPs in fish. The plausibility of these reports should be questioned, and cross-contamination should be considered.

Other studies working with relatively small particles have also reported the translocation of MPs to the liver. In a study using 0.5 µm MPs in *Oreochromis niloticus*^52^, the photomicrographs available to support the observations merely displayed fluorescence in the liver, while fluorescence was observed diffusely in the remaining organs, which is likely to be attributed to leaching of the fluorescent dye and not necessarily the presence of MP particles^53^. In another study with *D. rerio* using 5 µm MPs^48^, the general quality of the photomicrographs did not support the stated results, as previously pointed out by other authors^54^.

Even though the direct consequences of MP ingestion have been detailed (i.e., damage and physical blockage of the digestive system and limitation of food intake)^23^, our understanding of the impacts of MPs is still limited and often ambiguous. Tissue alterations have been described as a consequence of MP exposure. Pedà et al. reported that pristine MPs induced moderate to pronounced alterations of the distal intestine after 30 days of exposure in *Dicentrarchus labrax*^55^. Thinning of the bowel wall and epithelial damage were described by Qiao et al. in the intestine of *D. rerio* exposed for 21 days^56^. Similarly, Lei et al*.* showed that MP particles caused cracking of villi and splitting of enterocytes in *D. rerio*^57^. In the same species, Lu et al*.*^48^ reported signs of inflammation and lipid accumulation in the liver after exposure to MPs. In contrast, Asmonaitè et al*.* found limited impacts on gut integrity in *Oncorhynchus mykiss* exposed to MP particles for 4 weeks^58^. Jovanovic et al*.* concluded that dietary exposure of *S. aurata* for 45 days to different types of MPs did not cause any damage in the studied tissues^47^.

The ambiguity of the results obtained regarding the effects of pristine MPs in fish is largely due to a recurring problem of inaccuracy in the interpretation of the histopathological findings. Similar worries have been expressed by several veterinary pathologists. For instance, multiple errors have been detected in the aforementioned article by Lu et al.^48^ (see Comment on “Uptake and Accumulation of Polystyrene MPs in *D. rerio* (*Danio rerio*) and toxic Effects in Liver” by Baumann et al.^54^) as well as in an article by Deng et al.^59^ (see Uptake of MPs and related health effects: a critical discussion by Braeuning^50^). The problem with inaccurate data is that it will persist in the literature and will be unequivocally considered reliable. As a consequence, erroneous information will be unintentionally replicated and perpetuated by several authors.

In the present study, two separate experiments were performed using *Danio rerio*, a vertebrate model used for toxicological studies. The acute experiment was conducted to assess ingestion, intestinal retention time, uptake and elimination of MPs in *D. rerio* fed a single intake of pristine MPs. The sub-chronic experiment was designed to evaluate the potential for accumulation and translocation of different types of pristine MPs after prolonged dietary exposure and to determine the microscopic effects of such exposure.

## Methods

### MP characterization

Green fluorescent spherical microbeads (proprietary polymers of an undisclosed composition) with diameters of 1–5 µm were purchased from Cospheric LLC, USA (Supplementary Fig. S1). The microbeads were dissolved in ultrapure water (density: 1.3/cm^3^) to prepare stock solutions and were fluorescently labelled green with excitation and emission wavelengths of 515 and 414 nm, respectively. According to the manufacturer, the fluorescent particles were hydrophilic, and the addition of surfactant was not necessary. Fluorescent particles were used to enable the identification of small MPs (1–5 µm) in fish tissues.

Microfragments of plastic, labelled as polyethylene (PE), were obtained from a cosmetic body cleanser. The content of the cleanser was washed with distilled water and sieved to obtain particles ranging in size from 120–220 µm (mean: 175 ± 42 µm) (Supplementary Fig. S1). White nylon microfibres, obtained from a synthetic textile, were cut under a stereomicroscope to obtain fibres with an average width and length of 13.67 µm and 1.5 mm, respectively (Supplementary Fig. S1).

To determine the composition of the plastic polymers used, Fourier transform infrared (FTIR) spectroscopy was performed. A Bruker IFS 66/S spectrometer (Bruker, Spain) equipped with a deuterated triglycine sulphate (DTGS) detector and a diamond crystal attenuated total reflection (ATR) module was used. FTIR spectra were acquired from an average of 64 scans obtained with an 8 cm^-1^ resolution. The reflectance ratio (*R*/*R*_0_) was calculated, where *R* and *R*_0_ are the reflectances measured at the sample and the clean crystal, respectively. Positive bands represent the loss, while negative bands represent the gain of species at sampling. The cosmetic microfragments were confirmed to be PE (Supplementary Data S1). The spectrum obtained by the analysis of the fluorescent microbeads showed a slight similarity to that of polyethylene glycol (< 60%) but was insufficient to determine the polymer composition (Supplementary Data S2).

### Ethics approval

All the experimental protocols used in this study were revised and approved by the Animal Ethics Committee of the University of Las Palmas de Gran Canaria and authorised by the competent authority of the Canary Islands Government (Reference number: OEBA-ULPGC 23/2018). GPower3.1 software was used to determine the number of necessary animals per experimental group, with a statistical power of 0.95 and an alpha error of 0.05. All the protocols were designed and performed to result in the death of as few animals as possible and to reduce the duration and intensity of suffering, in accordance with the relevant guidelines and regulations (Directive 2010/63/EU).

## Experimental design

During the experiments, three petri dishes with ultrapure water were placed next to each work area and analysed as procedural blanks. The procedural blanks were present at every step of the MP evaluation process to assess sample contamination.

### Acute experiment: feeding behaviour, intestinal retention time, uptake and elimination

Seventy *D. rerio* adults of similar weight were purchased from Tropical Centre (ICA Canarias) and kept in acclimation tanks for 4 weeks to adapt to the laboratory conditions. The fish were placed in a semi-static system with tap water conditioned with JBL Biotopol and JBL Denitrol, according to the manufacturer’s instructions, at a stocking density of ~ 0.8 fish/dm^3^ (0.4 g fish/dm^3^) in the animal experimental facility (EGC00616436) at the University of Las Palmas de Gran Canaria. The fish were kept under a natural photoperiod of 12:12 h light:dark cycle. Water chemistry parameters were assessed every two days using a colorimetric test kit (nitrate, 10 mg/dm^3^; nitrite, 0 mg/dm^3^; pH, 6.8; total hardness, 80–300 mg/dm^3^; chlorine, 0 mg/dm^3^). Dissolved oxygen (> 6.0 mg/dm^3^) and temperature (23–25 °C) were also measured. The water was partly (60%) manually dumped, and debris (uneaten food and faeces) was siphoned from the bottom of the tanks.

During the acclimation period, fish were fed a control diet three times a day (20% of their body weight/day). The control diet was prepared using flake-shaped commercial food for aquarium fish (Basic, DAJANA PET, s.r.o.; 47% protein, 8% water, 7% ash, 5% fat and 2% fibres), which was ground into a fine powder. A gelatine leaf was melted in 27 °C water and mixed with the ground flakes. The final mix was refrigerated at 4 °C until it was solid.

A preliminary study was performed to assess the ability of *D. rerio* to recognize plastic particles as inedible material. Free fluorescent microbeads (0.328 g) were added to an aquarium with five *D. rerio*. Likewise, cosmetic microfragments (0.031 g) were added to another aquarium with the same number of fish. The fish from both groups were sampled 2 h post-feeding (hpf). Euthanasia and necropsies were performed as detailed below.

For the acute experiment, two sets of diets were used. Diet F_A_ was obtained by adding fluorescent microbeads (18.6 × 10^–2^ g/cm^3^, 16% of the total food delivered) to the control diet. Diet C_A_ was obtained by mixing cosmetic PE microfragments and textile microfibres (2.1 × 10^–2^ g/cm^3^, 2% of the total food delivered) with fish oil-aromatized gelatine. The gelatine was used as a medium to retain the plastic particles in the food and avoid their separation when incorporated into the water. Both diets were refrigerated at 4 °C until solid.

Following acclimation, the fish were starved for 24 h, randomly collected and then separated into two groups (80 dm^3^ per aquaria, n = 30). The fish in group 1 (0.333 ± 0.071 g) were fed a single intake of diet F_A_. The sum of the fluorescent MPs provided accounted for 3% of the fish body weight. The fish from group 2 (0.360–0.063 g) were fed a single intake of diet C_A_, which accounted for 0.3% of the fish body weight. In both cases, feeding took approximately 30 min. Following this period, to prevent food contamination, the fish from both groups were separated into five aquaria in groups of six. Each aquarium corresponded to a different sampling time (2, 6, 10, 12 and 24 hpf). The fish were closely monitored during these procedures. At every sampling point, the water from each aquarium was filtered, and faeces were recollected and mounted on a slide.

The fish from group 1 and group 2 were sampled 2, 6, 10, 12 and 24 hpf. To avoid contamination, each group had its own handling equipment. Euthanasia was achieved by anaesthetic overdose (0.5–0.6 cm^3^/dm^3^) through immersion in 2-phenoxyethanol (Sigma-Aldrich). Necropsies were performed under a stereomicroscope (Motic SMZ-161 TL, China). The whole intestine was extracted and fixed in 10% neutral buffered formalin for histology.

### Sub-chronic experiment: translocation and toxicity

Seventy-two *D. rerio* adults of similar weight and length were purchased from the same supplier and kept in acclimation tanks under the same conditions as those described for the acute experiment.

Two sets of experimental diets were used. Both were prepared the same way as the control diet to ensure homogeneity throughout the test food. Each test diet was spiked with different types of MPs. Diet F_SC_ was obtained by adding fluorescent microbeads (9.9 × 10^–4^ g/cm^3^, 0.1% of the total food delivered) to the control diet. Likewise, diet C_SC_ was obtained by mixing cosmetic PE microfragments and textile microfibres (3.3 × 10^–2^ g/cm^3^, 3% of total food delivered) with the control diet. A control group, held under identical conditions, was fed a control diet.

After the acclimation period, the *D. rerio* individuals were weighed, and their general body shape and urogenital papilla were inspected to determine their sex. The fish were distributed into two test groups. Fish from group 1 (0.427 ± 0.04 g) were fed the F_SC_ diet, and fish from group 2 (0.568 ± 0.112 g) were fed the C_SC_ diet. All the treatments were carried out in triplicate, and each aquarium comprised an equal number of males (n = 4) and females (n = 4). Each batch (n = 8) was placed in a 20 dm^3^ aquarium. A control group (n=24) was added.

The fish from all the groups were fed a control diet three times a day (20% of their body weight) on a fixed schedule. Every two days, the first intake of the control diet was replaced with the F_SC_ diet (0.01% MPs/fish/day) and C_SC_ diet (0.2% MPs/fish/day) in the test groups.

After the second week, the fish from the cosmetic PE group started to manifest anorexia and impaired reactivity to stimuli. The control diet was then reduced to two intakes a day for a week to evaluate the fish response. After a week, the initial feeding routine was resumed.

During feeding time, the fish were monitored for behavioural changes. The feeding experiment lasted 45 days. Mortalities and observable abnormalities regarding both appearance and behaviour were recorded. All the aquaria contained tap water under constant aeration. The filters were regularly washed, and clean water was added.

After 30 and 45 days of feeding, two fish from each replicate aquarium were euthanized. Euthanasia was performed as described for the acute experiment. The weight of each fish was recorded. To prevent contamination with particles, a cut was made in the ventral line. The whole fish were fixed in 10% neutral buffered formalin for 24 h. Whole intestine, liver and muscle sections were then extracted under a stereo microscope (Motic SMZ-161 TL, China). The fish from the control group were similarly dissected. Contamination was prevented and confirmed by the absence of MPs in the control group.

The depuration period was designed to determine the potential for the bioaccumulation of these plastic particles. At the end of the feeding period, the remaining fish were transferred to new aquaria to avoid contamination with the test substance. For 15 days, all the fish were fed only the control diet. At the end of the depuration period, two fish per triplicate were euthanized, weighed and similarly dissected.

### Histology

The formalin-fixed tissues were dehydrated, cleared and embedded in paraffin and sectioned at 4 μm. The obtained samples were stained with haematoxylin and eosin (H&E)^61^. Five sections were made from each sample. The slides were mounted and examined with a light microscope (Olympus BX51TF, Japan).

For the assessment of the histological findings in the intestine and liver, the methodology proposed by Saraiva et al*.*^62^ and Bernet et al*.*^63^, respectively, was followed. For each functional unit (i.e., the liver tissue, interstitial tissue and bile duct for the liver and the epithelium and lamina propria for the intestine), pathological changes were classified into five reaction patterns: circulatory disturbances, regressive changes, progressive changes, inflammation and tumour development. The alterations (w) were classified into three important factors: minimal pathological importance (1) if easily reversible, moderate pathological importance (2) and marked pathological importance (3) if generally irreversible. Additionally, a score value (a) ranging from 0 to 6 was used for every alteration, depending on the degree and extent of the alteration: unchanged (0), minimal (1), mild (2), mild to moderate (3), moderate (4), marked (5) and severe (6). Mathematical calculation of lesion indices was performed to assess the degree of damage for each organ separately. An individual description, termed *degree of vacuolation,* was used in addition to the alteration classifications of the reaction patterns. The degree of vacuolation was scored for all the fish using a semiquantitative scale: minimal (1), mild (2), mild to moderate (3), moderate (4), marked (5) and severe (6). For the index calculations, these were not considered, since the changes were already covered by the standardized expressions (plasma alterations; decreased hepatocellular vacuolation) within the respective reaction pattern (Supplementary Tables S1 and S2).

### Confocal microscopy

Confocal microscopy was used to assess the presence and uptake of fluorescent MPs by the different tissues. Fluorescence images were acquired with a confocal microscope (Zeiss Confocal LSM800, Germany) at an excitation wavelength of 519 nm and emission wavelength of 543 nm for green and an excitation wavelength of 543 nm and an emitting wavelength of 567 nm for orange. Panoramic images of the whole *D. rerio* intestine were created. To confirm the internalization of the MP particles in tissues, a series of two-dimensional images over the depth ranges of interest (Z-stacks) were performed to obtain a three-dimensional image. The diameter of the microparticles was measured using Zen Blue v2.3 software.

### Statistical analysis

For comparisons between groups, Wilcoxon’s tests or Student’s t-tests were used, and the results are presented as the mean (standard deviation) or median [range]. Differences were considered significant when the two-tailed P value was below 0.05. The statistical analyses were performed by a commercial statistical software package (IBM SPSS Statistics Version 18, SPSS Inc., Chicago, IL).

## Results

### Acute experiment

#### Feeding behaviour

When exposed to free cosmetic PE MPs, *D. rerio* displayed avoidance behaviour towards food. Two distinct manifestations of avoidance were recorded. In the first case, some fish immediately recognized the MP particles as inedible materials, turning or passing the particles and avoiding contact. In the second case, fish initially moved towards the particles, capturing and nibbling them before spitting them out. In both cases, the fish recognized MP particles as inedible elements.

Occasional fluorescent MPs were observed in the intestinal lumen of the fish when the histological sections were assessed. These observations hint at the possibility of accidental ingestion enabled by the small size of the particles (1–5 µm).

When the MPs were blended with either commercial food (diet F_A_) or fish oil (diet C_A_), *D. rerio* actively displayed a prey capture behaviour, identifying the food, either visually or by chemosensation, tracking it, with a series of routine bends, capturing it and finally ingesting it.

#### Intestinal retention time, uptake and elimination

**Figure 1:**
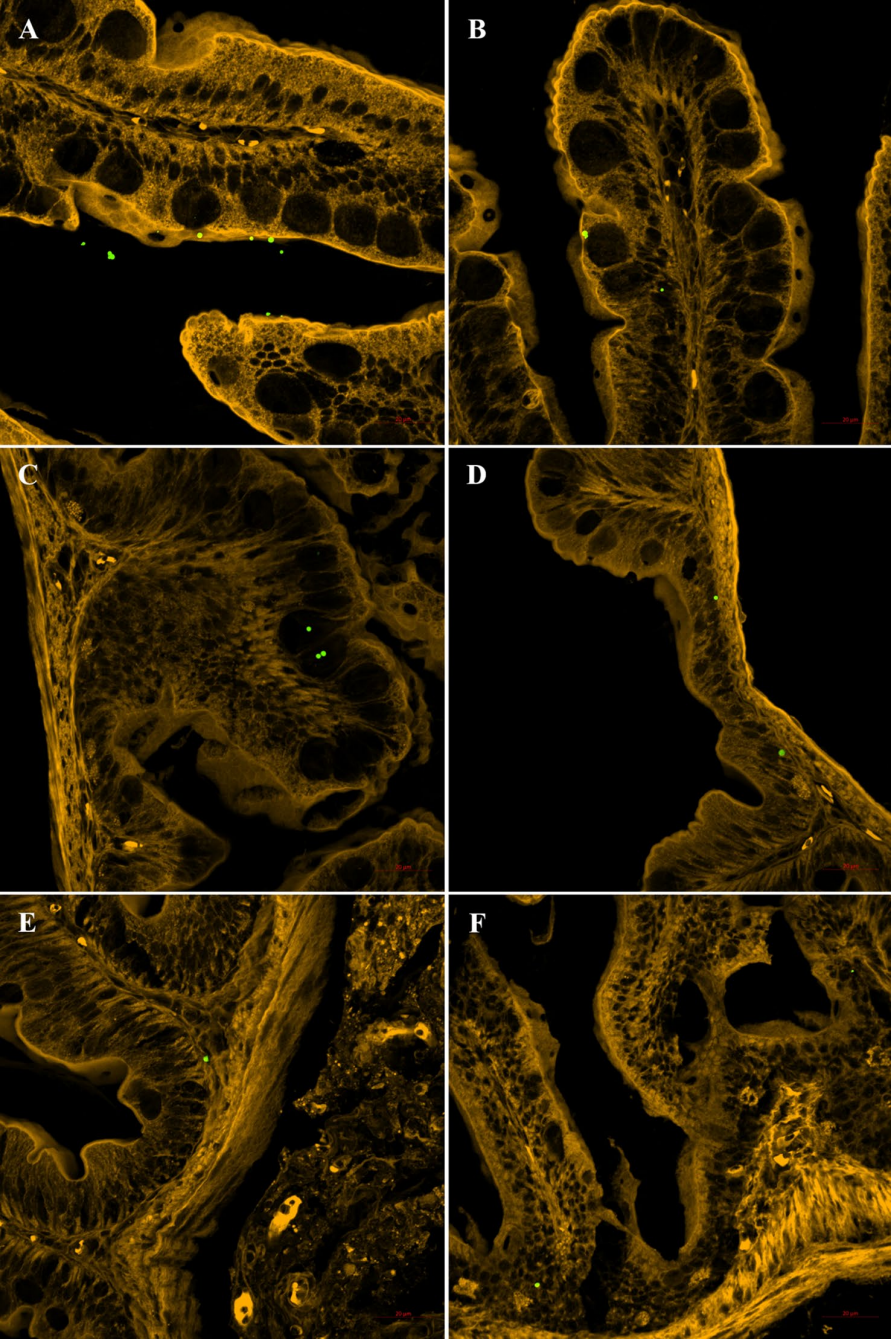
Intestinal segments under confocal microscopy. (**A**) Two hours post-feeding: fluorescent particles in contact with the microvilli on the apical surface of the enterocytes and (**B**) on the apical and basal aspects of the enterocyte cytoplasm. (**C**) Six hours post-feeding: particles inside the goblet cells. (**D**) Ten hours post-feeding: particles in the cytoplasm of the enterocyte and (**E**) in the lamina propria. (**F**) Twelve hours post-feeding: particles within the lamina propria (scale bar = 20 µm).

Two hours after the ingestion of diet F_A_, 67% of all the fish presented a high number of fluorescent MPs in the lumen of the mid-intestine (Supplementary Fig. S2). Under confocal microscopy, the fluorescent MPs were observed in the villi and in the apical surface of the enterocytes (Fig. 1A). An average of two to three particles per fish was present in both the apical and basal aspects of the enterocyte cytoplasm (Fig. 1B, Supplementary Fig. S2). At 6 hpf, 67% of the fish had fluorescent MPs in the mid- and posterior intestine (Supplementary Figs. S2 and S3). Most fluorescent particles were admixed with the digestive content. Fluorescent MPs were observed inside the goblet cells in 100% of the fish (Fig. 1C, Supplementary Fig. S2). At 10 hpf, fluorescent MPs were present in the lumen of the posterior intestine, admixed with the digestive content (Supplementary Fig. S2). MPs were detected in the enterocyte cytoplasm (Fig. 1D) and inside the goblet cells in 100% of the fish, similar to our observation at 6 hpf. Particles were found in the lamina propria (Fig. 1E) in 67% of the fish (Supplementary Fig. S2). At 12 hpf, most of the fluorescent MPs were in the final portion of the posterior intestine (Supplementary Figs. S2 and S3). In 33% of the fish, particles were visualized within the lamina propria (Fig. 1F, Supplementary Fig. S2). Twenty-four hours after intake, fluorescent MPs were not detected in either the digestive content or the intestinal mucosa (Supplementary Figs. S2 and S3). Overall, the particles that crossed the epithelial barrier had a size ranging from 1.083 to 2.041 µm. The Z-stack photos confirmed the internalization of the MP particles in the cytoplasm of the enterocytes and in the lamina propria (Supplementary Figs. S4–S6). Fluorescent MPs were found in the faeces of the fish 6 hpf and were evident between 6 and 12 hpf, decreasing after 24 hpf (Supplementary Fig. S7).

**Figure 2:**
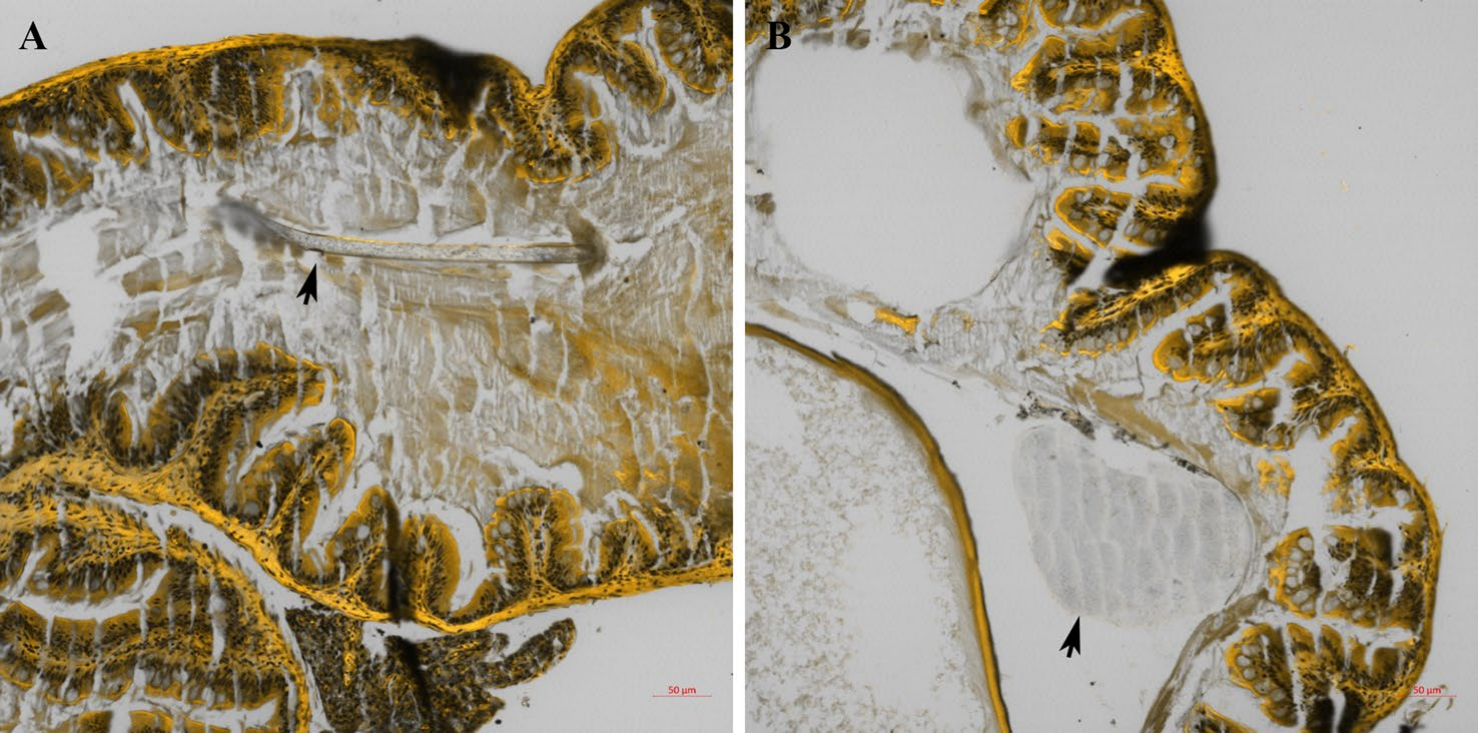
Intestinal segments under confocal microscopy. Twenty-four hours post-feeding: (**A**) synthetic microfibres (arrow) and (**B**) cosmetic PE microplastics in the last segments of the intestine (arrow) (scale bar = 50 µm).

Cosmetic PE microfragments and microfibres were found in the intestinal lumen of the fish fed the C_A_ diet. At 2 hpf, 67% of the fish had microfragments and microfibres in their lumen of the mid-intestine (Supplementary Fig. S2). From 6 to 12 hpf, microfragments were detected in the lumen of the middle and posterior segments of the intestine (Supplementary Figs. S2 and S3). At 24 hpf, cosmetic microfragments and microfibres were observed in the last segments of the intestine (Fig. 2, Supplementary Figs. S2 and S3). An average of 15 particles were detected in the faeces collected between 10 and 24 hpf (Supplementary Fig. S8). Cosmetic microfragments and microfibres did not cross the intestinal barrier (Supplementary Fig. S2).

### Sub-chronic experiment

#### Survival rate, weight gain and feeding behaviour

Mortalities were recorded for all the test and control groups. Survival rates were 100% in all the test groups. For the control group, the survival rate was 95.8%, as a single fish (1/24) died four weeks after the beginning of the experiment.

Regarding weight gain (weight gain (%) = final wet weight-initial wet weight gain), no significant differences were seen among the different groups after 45 days.

For the first two weeks, once visual contact with food was established, in a sign of recognition, fish from all the test groups displayed an aggregating behaviour towards the food, approaching, capturing, nibbling and chewing the food particles. However, after the second week, the fish in all the replicates fed the C_SC_ diet started to exhibit avoidance behaviour towards food. Anxiety-like behaviours, such as anorexia, hypoactivity and reduced exploration, under-reactivity to stimuli, and a tendency to display an abnormal distribution on the bottom of the tank, were recorded. Additionally, a general change in body colour (i.e., a lightened skin pigmentation) was observed in most animals. Food deposition at the bottom of the aquaria was additionally observed.

After reducing the number of intakes of the control diet for a week, the appetite and activity of the fish fed the C_SC_ diet significantly improved. However, despite their improvements, the fish did not fully return to their initial state of responsiveness. Mild hypoactivity and under-reactivity to stimuli were observed over time. No other clinical signs were observed throughout the experiment.

#### Particle retention and translocation

**Figure 3:**
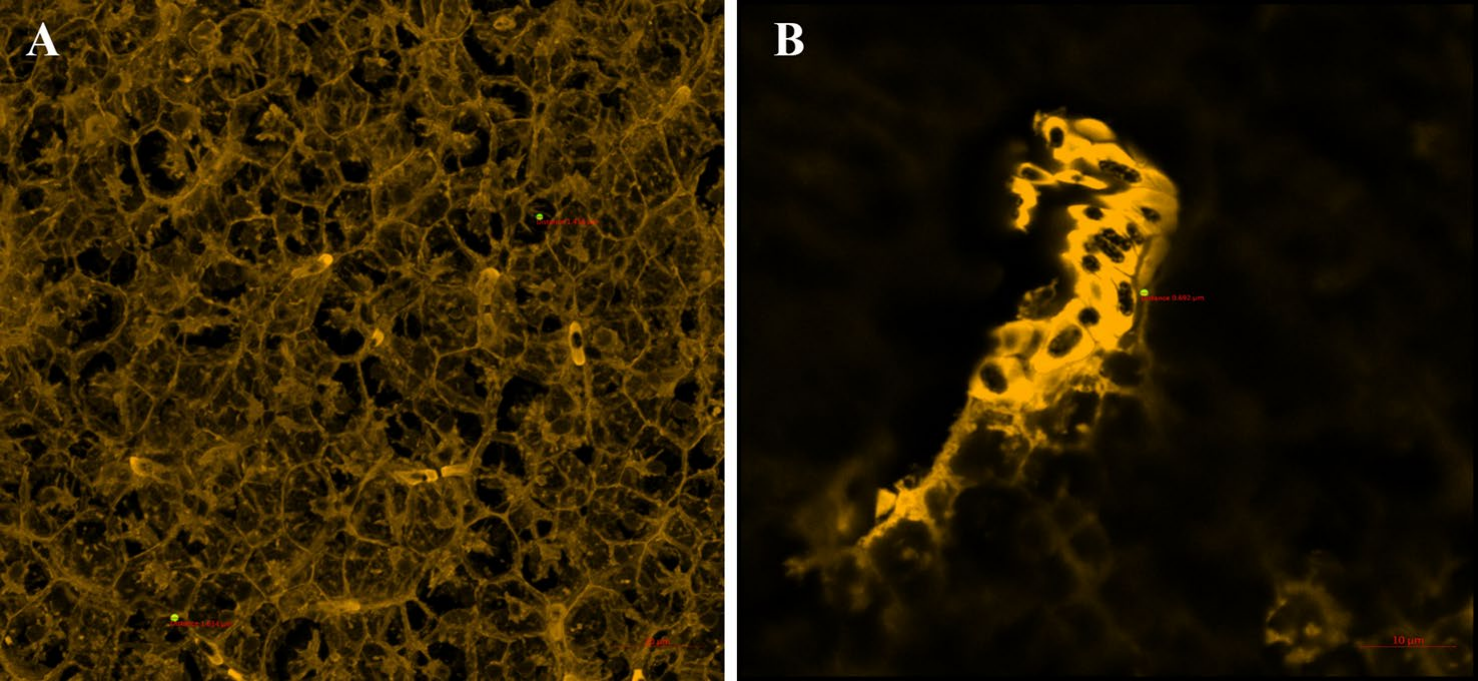
Liver under confocal microscope. (**A**) Fluorescent microspheres within the cytoplasm of the hepatocytes (scale bar = 20 µm) and (**B**) surrounding a blood vessel (scale bar = 10 µm).

Fluorescent MPs were observed in 16.7% (2/12) and 33% (4/12) of the livers of the fish fed the F_SC_ diet thirty and forty-five days after the beginning of the experiment, respectively. MPs particles found in the cytoplasm of the hepatocytes ranged between 1.416 and 1.634 µm (Fig. 3A). A single particle (0.692 µm) was also observed surrounding a blood vessel (Fig. 3B). Serial Z-sections of the liver confirmed the internalization of the MP particles (Supplementary Fig. S9). Fluorescent MPs were not detected in the muscle.

MP particles were not found in any organ apart from the intestinal lumen in the fish fed the C_SC_ diet.

The presence of MPs after 15 days of depuration was also assessed. Fluorescent particles were not observed in the intestinal tract after the depuration period, whereas a small number of cosmetic particles (average 3.3 particles) were detected in the intestinal tract in 50% of the fish (Supplementary Fig. S10). Apart from the intestine, MP particles were not observed in any other tissue.

MPs were not found in the procedural blanks set up to assess contamination.

#### Histopathological changes

**Figure 4:**
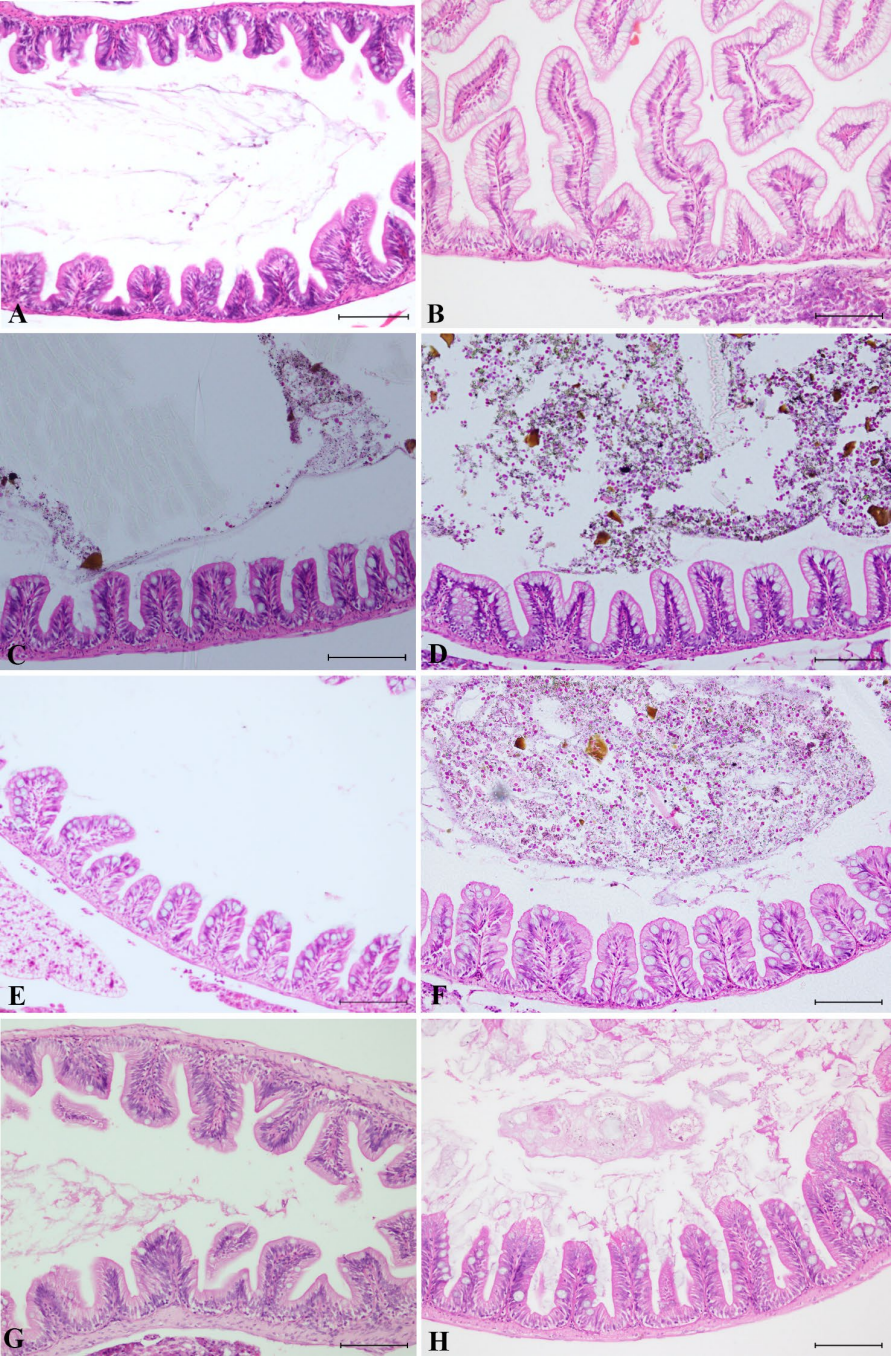
Intestine: (**A**, **B**) control, (**C**, **E**, **G**) cosmetic and (**D**, **F**, **H**) fluorescent groups. (**A**) Normal histology of the mid-intestine. (**B**) Specialized enterocytes with supranuclear vacuoles in the posterior region of the mid-intestine. Thirty days after the start of the experiment: intestinal sections from fish fed (**C**) cosmetic MPs and (**D**) fluorescent MPs. Forty-five days after the start of the experiment: intestinal sections from fish fed (**E**) cosmetic MPs and (**F**) fluorescent MPs. Post-depuration: intestinal sections from fish fed (**G**) cosmetic MPs and (**H**) fluorescent MPs (scale bar = 100 µm, **H**–**E**).

Intestinal samples were assessed following the guidelines proposed by Saraiva et al*.*^62^ as follows (Supplementary Table S1, Fig. 4).

Regarding regressive changes, among the epithelial cells, occasional scattered individual apoptotic cells were observed in fish from all the groups. However, such observations were regarded as McKnight cells, which are often seen in healthy fish in low numbers^64^.

Inflammatory changes were not observed, and the number and size of goblet cells did not show any significant variation. For the remaining reaction pattern groups, changes were not observed. Overall, compared with the control group, pathological changes were not observed in any of the test groups during the experimental period.

**Figure 5:**
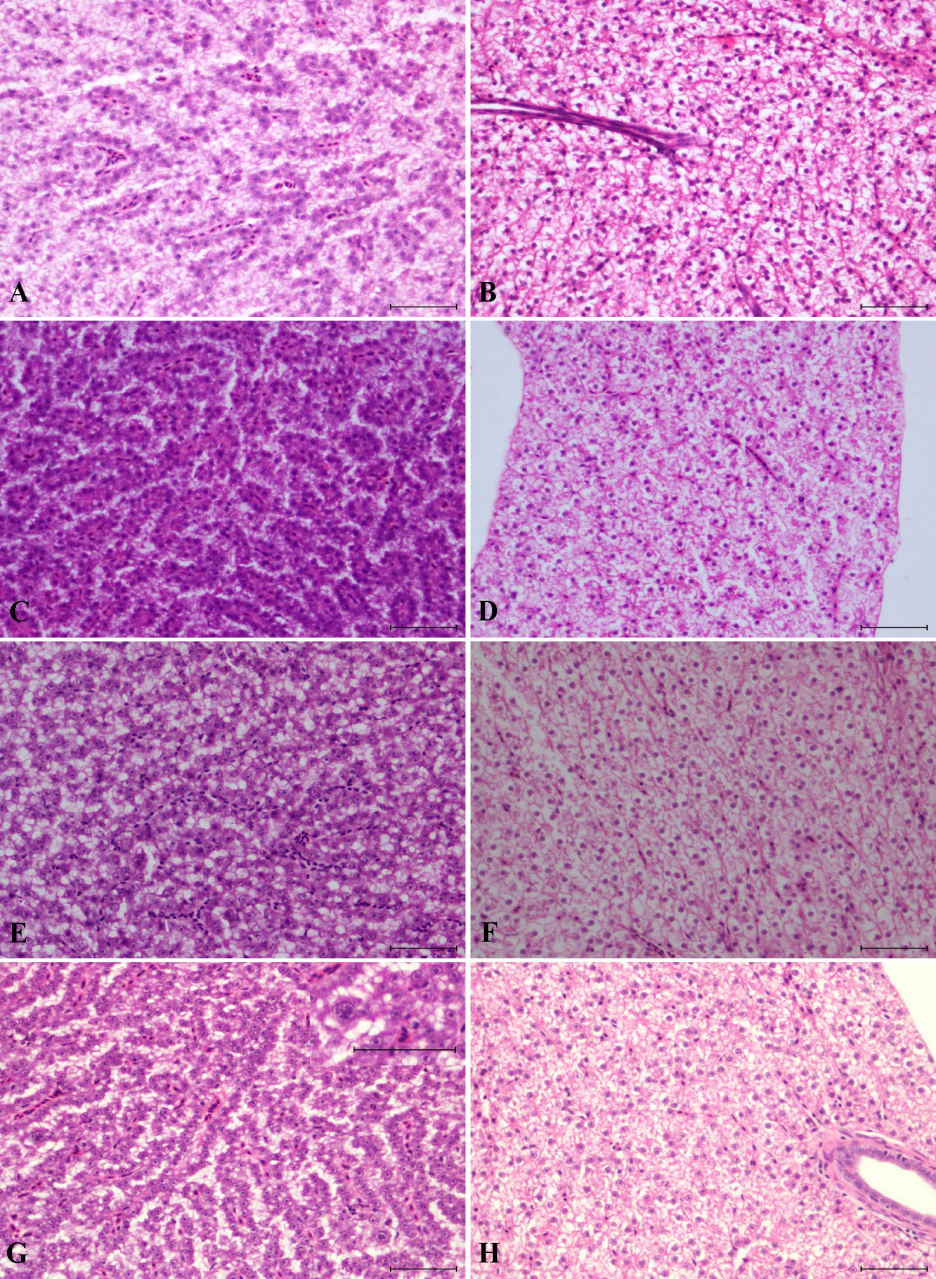
Liver sections from fish fed fluorescent MPs. Control livers were sampled thirty days after the beginning of the experiment from (**A**) a female showing mild to moderate vacuolation and (**B**) a male with mild to moderate vacuolation. Thirty days after the start of the experiment: (**C**) female with minimal vacuolation and (**D**) male with mild to moderate vacuolation. Forty-five days after the start of the experiment: (**E**) females with mild vacuolation and (**F**) females with mild to moderate vacuolation. Post-depuration: (**G**) female with mild vacuolation, showing occasional nuclear enlargement (karyomegaly) (see inset, scale bar = 50 µm), and (**H**) male with mild vacuolation (scale bar = 100 µm, **H**–**E**).

**Figure 6:**
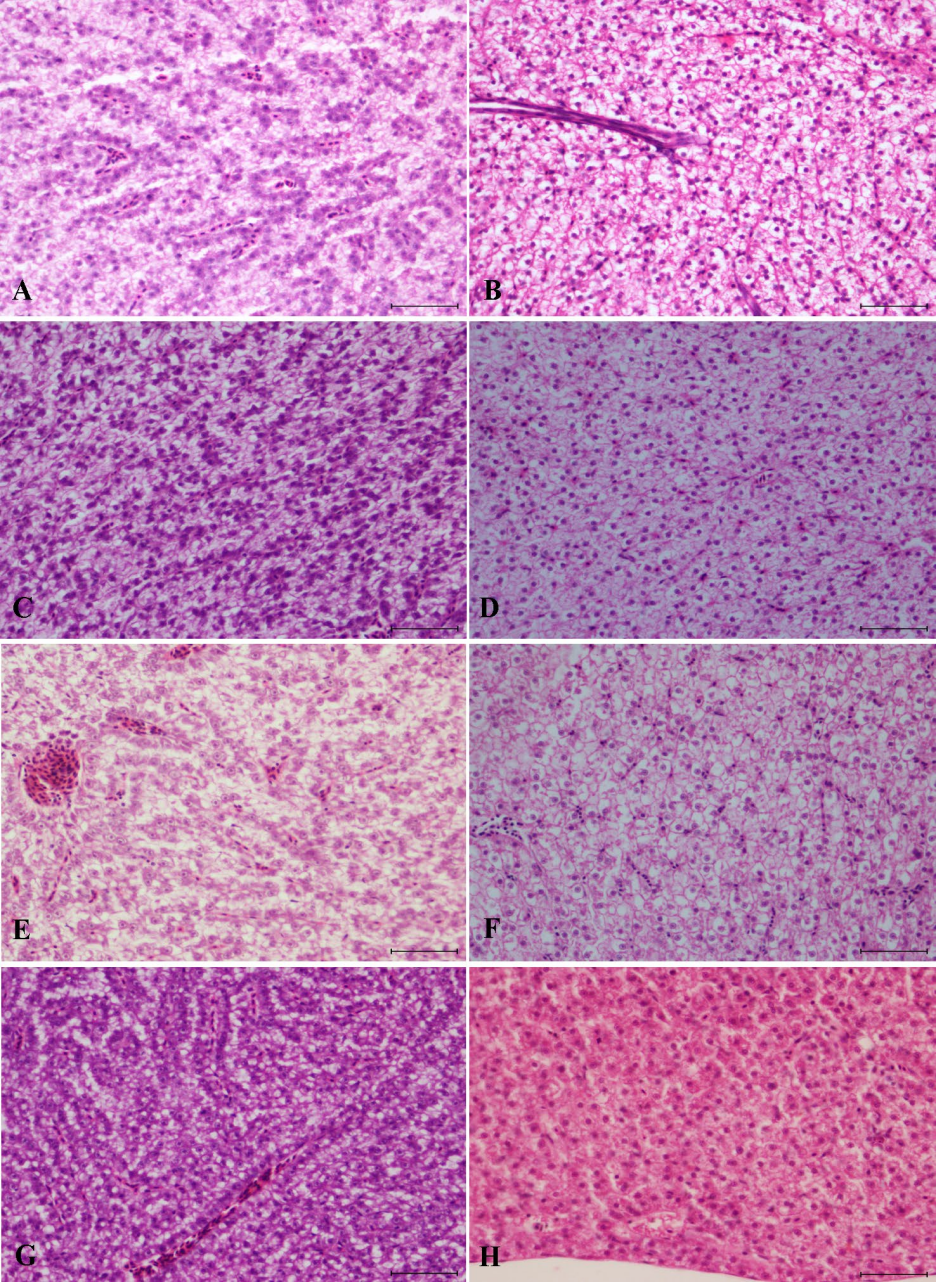
Liver sections from fish fed cosmetic MPs. Control livers were sampled thirty days after the beginning of the experiment from (**A**) a female showing mild to moderate vacuolation and (**B**) a male with mild to moderate vacuolation. Thirty days after the start of the experiment: (**C**) females showing moderate vacuolation and (**D**) males with mild to moderate vacuolation. Forty-five days after the start of the experiment: (**E**) female presenting mild to moderate vacuolation and (**F**) male showing mild vacuolation. Post-depuration: (**G**) female liver with mild vacuolation and (**H**) male with minimal vacuolation (scale bar = 100 µm, **H**–**E**).

The liver samples were evaluated according to the guidelines by Bernet et al.^63^ as follows (Supplementary Table S2, Figs. 5 and 6).

Regressive changes involving plasma alterations in the liver were noted, particularly vacuolar degeneration. However, decreases in vacuolation were also observed. For that reason, an additional description pattern, termed *degree of vacuolation*, was added. The scoring of this pattern was obtained by comparing both test groups with the corresponding control groups. In the control group, livers from females presented hepatocytes with mottled cytoplasm, a deeply basophilic appearance in the peri-sinusoidal regions and mild to moderate enlargement due to lipid-like vacuolation (score: 3) (Figs. 5A and 6A). On the other hand, males from the control group showed primarily round to polygonal hepatocytes with clear cytoplasmic vacuoles containing slightly flocculent material with soft margins and centric to paracentric nuclei (score: 3) (Figs. 5B and 6B).

For the test groups, overall, 22.2% (4/18) of the females had minimal to mild vacuolation (score: 1–2) (Fig. 5C, E), while 27.8% (5/18) had moderate vacuolation (score: 4) (Fig. 6C, E). Additionally, 27.8% (5/18) of the males displayed both minimal and mild vacuolation (score: 1–2) (Figs. 5H and 6H), with the remaining males presenting a similar degree of vacuolation as that in the control group (score: 3) (Figs. 5D, F and 6D, F).

When comparing the test groups, in the group fed fluorescent MPs (Figs. 5), minimal to mild vacuolation (score: 1–2) was observed in both females (33.3%) and males (22.2%) (3/9 and 2/9, respectively). A total of 22.2% (2/9) of the females showed moderate vacuolation (score: 4) and no observable changes were recorded in the males. In the cosmetic test group (Fig. 6), 11.1% (1/9) and 33.3% (3/9) of the females and males, respectively, showed minimal to mild vacuolation (score: 1–2). Moderate vacuolation (score: 4) was observed in 44.4% (4/9) of the females, and no observable differences were noted in the males. Over the duration (forty-five days) of the experiment, 33.3% (4/12) of the females presented moderate vacuolation (score: 4), while 25% (3/12) presented minimal to mild vacuolation (score: 1–2). The males did not show differences compared to the control group. However, after the period of depuration, 33.3% (2/6) and 83.3% (5/6) of the females (Figs. 5G and 6G) and males (Figs. 5H and 6H), respectively, presented minimal to mild vacuolation (score: 1–2).

Histologically, moderate vacuolation in both sexes translated into an enlargement of the hepatocyte cytoplasm due to the presence of one or several vacuoles that displaced the nuclei to the periphery. In contrast, when minimal to mild vacuolation was observed, the hepatocytes appeared smaller, and the amount of basophilic and eosinophilic material in the hepatocyte cytoplasm was predominant when compared to the smaller space occupied by the optically empty and irregular vacuoles. Additionally, the nuclei were rounded and centrally located.

Occasional pyknotic-like hepatocyte nuclei were observed in 100% of the fish in all the groups, including the control group. These findings were attributed to suboptimal fixation of the livers. Pyknosis seldom occurs as an isolated finding and is usually accompanied by other evidence of hepatocyte degeneration or necrosis, such as nuclear karyorrhexis or cytoplasmic hypereosinophilia^65^. Randomly distributed hepatocytes with nuclear enlargement (karyomegaly) (Fig. 5G) were observed in females in both treatment groups as well as in the control group. For that reason, these observations, previously observed in the livers of untreated *D. rerio*, were regarded as an idiosyncratic finding^65^.

No further changes were observed.

## Discussion

Propelled by wind, heavy rainfall and tidal currents, MP contamination^12,66,67^ has spread to remote lakes^19,68^, rivers^14,21,69–71^, estuarine regions^72–74^, seas^41,75^, oceans^76^ and even sea ice^77^. As a result, a great number of species are currently at risk of exposure and susceptible to ingestion of MPs^8,10,25–28^, either through dietary exposure or by transfer along the food chain.

The predominant types of MPs found in aquatic systems are fibres^14–16,44^, microfragments^6,7,19,20^ and microbeads^21^. In previous studies, the type of MPs^78,79^ as well as shape^80^ and size were suggested to influence the level of toxicity inflicted on fish tissues. To replicate those observations, different types (fibres, fragments and beads), shapes (irregular and regular) and sizes of MPs (1–255 µm) were used in the present study.

### Acute experiment

#### Feeding behaviour

*Danio rerio* were offered free MPs to determine their ability to recognize plastics as inedible particles, as previously suggested by Kim et al.^81^ Following exposure, all the fish displayed a clear refusal behaviour, suggesting that *D. rerio* knowingly recognized plastic as inedible particles. Kim et al*.*^81^ detailed that capture events in *D. rerio* had the lowest rates when fish were exposed only to MPs. Most plastic particles were also quickly rejected in a study by Colton et al*.*^82^ However, despite recognizing plastic as an inedible element, several authors have documented the presence of MPs in the gastrointestinal tract (GIT) of fishes^83–87^.

Accidental consumption when foraging on aggregated prey^46^ has been observed. Additionally, visual cues that resemble prey, such as colour or shape, may enable the ingestion of smaller particles, hindering the distinction between prey and plastic particles^22,46,88^. Likewise, it has been suggested that odours associated with biofouled plastic debris stimulate foraging behaviour^81,89^.

In our study, we observed that free smaller fluorescent MPs were occasionally present in the intestinal lumen. It seems that due to their small size (1–5 µm), these fluorescent MPs were unintentionally ingested. In contrast, larger particles of cosmetic MPs were not observed inside the intestine of *D. rerio*, and accidental ingestion was excluded. Our observations seem to suggest that smaller MPs are more difficult to differentiate from normal prey as previously reported by Critchell & Hoogenboom^22^ and/or that low-density MPs of a smaller size are more likely to be passively ingested while gulping air^46,90^.

When MPs were mixed with commercial fish food (diet F_A_) and fish oil (diet C_A_), ingestion rates greatly increased. These observations support previous reports that chemical cues resembling prey^89^ and that higher food concentrations are likely to increase MP ingestion^46^.

#### Intestinal retention time, uptake and elimination

The uptake of MP particles was observed in *D. rerio* fed a single meal of the F_A_ diet. Fluorescent microbeads were detected in the apical and basal surfaces of the enterocytes, inside the goblet cells and in the lamina propria. The internalization of these particles was confirmed by the Z-stack sections. However, despite the uptake of several particles, after 24 h, the MPs had completely cleared from the GITs of *D. rerio* without translocation to other organs. Penetration of individual particles (up to ~ 70 µm) into the goblet cells has been reported in other species^91^. Endocytosis of luminal material by goblet cells has been described^92^, and it has been suggested that some sub-populations of goblet cells may have relatively loose junctions^93^. In a study by Batel et al*.*, the uptake of a few particles was observed in 7.7% of *D. rerio*^24^*.* However, no further tests were performed to confirm the internalization of the particles. Additionally, the reported incidence was very low.

Conversely, *D. rerio* fed a single intake of the C_A_ diet presented plastic particles in only the intestinal lumen, and uptake was not observed. In this case, the uptake of microfragments and microfibres seemed unlikely due to their large size. After 24 h, only a microfibre and microfragment remained in the posterior intestine of a single *D. rerio*, indicating a short retention time for these MPs. Similar observations were described in *C. auratus*^45^ and *Cyprinodon variegatus*^80^ exposed to 5–200 µm and 6–350 µm particles, respectively.

In our study, the retention rates for MPs and food were similar. However, the retention rate for the fluorescent MPs (1–5 µm) was shorter than that for the cosmetic MPs, as after 24 h, all the fluorescent particles had been completely excreted. Despite their larger size and irregular shape, the cosmetic PE microfragments and microfibres were successfully eliminated in most fish after 24 h. The retention rates for the MPs in our study were similar to those observed by other authors. *D. rerio* was shown to rapidly excrete MP particles (70 nm–20 µm), reaching a steady state 48 h after exposure^48^, and *C. auratus* eliminated 90% of the particles (50–500 µm) to which it was exposed after 33.4 hours^45^. Similarly, *S. aurata* showed a retention rate close to zero, as 90% of the fish had cleared the MPs (~ 75 µm) after 24 hours^47^. Despite the similarities, larger particles are likely to take more time to be eliminated. *Seriolella violacea* fed MPs (length, 1.2 ± 0.2 mm; diameter, 1.0 ± 0.1 mm) took an average of 10.6 ± 2.5 days to egest the last MPs^46^. Furthermore, a study by Santos & Jobling performed on *Gadus morhua* fed a single meal with plastic beads (5,000 µm) and MPs (2000 µm) showed a delay in the evacuation of the 5,000 µm beads when compared to the time it took to egest the 2000 µm MPs^94^.

### Sub-chronic experiment

#### Survival rate, weight gain, and feeding behaviour

After sub-chronic dietary exposure to MPs, the survival rates were 100% in all the test groups. For the control group, the survival rate was 95.8%, as a single fish died at the beginning of the sub-chronic experiment. Our results are in line with previous observations in *D. rerio* exposed to MPs over two^95^ and 3 weeks^48^. Conversely, a significant reduction in survival rates was observed in *D. rerio* fed 10 mg/L PP MPs; the fish in this experiment additionally presented swollen abdomens^57^.

No significant weight differences were observed between the test groups and the control group. Similar results were reported in *Symphysodon aequifasciatus*^96^ and *S. aurata* exposed to MPs for 30 days^97^ and 45 days^47^. Likewise, *Acanthochromis polyaccantus* exposed to MPs (average 2 mm diameter) for 42 days did not show significant changes in body condition^22^.

After the second week, the fish fed the C_SC_ diet displayed anxiety-like behaviours, such as anorexia and lethargy, and lightened skin pigmentation. Decreases in feeding and swimming activity were reported in *Sebastes schlegelii* after exposure to PS MPs^98^ and *Cyprinodon variegatus* after exposure to irregular PE MPs^80^. Lethargy and paling can originate from several factors, such as infections, toxicity, environmental stress, oxygen depletion and starvation^99^. It was also hypothesized that this decreased response occurred upon repeated exposure to MPs and would be the result of habituation to such particles^100^. As in another study with *D. rerio* fed twice a day with nauplii loaded with high concentrations of MPs, there were no observable signs of stress or disease. However, in this case^24^, the ingested particles were relatively small (1–20 µm), which could explain the absence of signs of distress after the ingestion of multiple meals with MPs.

#### Particle retention and translocation

For the F_SC_ diet, fluorescent MPs accounted for 0.1% of the total ingested feed, similar to estimations by Jovanović et al.^47^ For the C_SC_ diet, the average density of MPs used was 2,137 items/m^3^ (2.49 particles/L). Despite the apparent high number of MP items used, similar or even higher values have been reported in aquatic environments^17,69,101^.

In addition, it has been reported that most approaches to estimate the numbers of MPs in aquatic ecosystems lead to underestimations^23^. A common problem with most field studies is that each study uses different mesh sizes, thus having a different cut-off size for the MPs analysed^23,102^.

After successive meals of the F_SC_ diet, followed by a period of depuration, *D. rerio* eliminated the particles ingested, as no trace of particles was found in the intestinal tract after the depuration period. In contrast, 50% of the fish fed successive meals of the C_SC_ diet retained an average of 3.3 particles/fish after the end of the experiment. The number of particles found in the intestine after multiple meals is consistent with that found in field studies, in which an average of 0 to 3 particles^34,36–44^ was documented. The low number of particles found in the digestive tract may be indicative of the short residence time of MPs within the GITs of fish. These results also appear to indicate that MPs are unlikely to accumulate within the intestine of fish over successive meals**.** However, particle size is clearly a determining factor when considering clearance rates for plastic particles. *G. morhua* fed multiple meals showed a longer retention rate, as the gastric half-life of the beads was substantially increased with particle size^94^. Our results support the observations by Santos and Jobling^94^ that MP retention rate seems to increase with increasing particle size and the intake of additional meals.

Nevertheless, it is important to bear in mind that the structure of the digestive tract varies among fish species^49^. Additionally, other intrinsic (e.g., genetic background, species, age, and physiology) and extrinsic (e.g., habitat, food, type of MPs, and methodology) factors have to be taken into account when discussing clearance rate patterns.

In our study, an average of 1.6 particles/fish was observed in the liver after successive meals of the F_SC_ diet. To validate the process of translocation, the internalization of these particles (up to 1.634 μm) was confirmed by multiple Z-stack sections. The translocation of MP particles to other tissues, such as the liver^47,50,51^ and muscle^37,47^, has been previously described. Avio et al*.* reported the translocation of plastic particles (200–600 μm) from the digestive tract to the liver in *M. cephalus*^50^. Likewise, Collard et al. documented the presence of two particles (39–90 μm) in the livers of *E. encrasicolus*^51^, and Abbasi et al*.* detailed the presence of variably sized MP particles (up to 250 µm) in the livers of four fish species captured in the Persian Gulf^37^. Similarly, Jovanovic et al*.* observed the presence of < 1 particle (214 ± 288 μm) in the liver of *S. aurata*^47^, carefully pointing to the possibility of cross-contamination. The alleged translocation of such large particles is difficult to explain with the current knowledge on translocation pathways for MPs in fish; thus, the plausibility of these reports should be questioned.

Other studies working with smaller particles have also reported the translocation of MPs to the liver^48,52^. A study exposing *O. niloticus* to 0.5 µm MPs allegedly observed the translocation of MPs to the liver^52^; however, the photomicrographs used to support those observations barely show fluorescence in the liver, and the fluorescence in the remaining organs is diffusely spread in the tissues. This is likely to be attributed to leaching of the fluorescent dye and not necessarily the presence of MP particles^53^. In another study with *D. rerio* using 5 µm MPs^48^, the photomicrographic evidence was inaccurate or poorly presented, as previously highlighted in another publication^54^.

As previously declared by Jovanović et al*.*^47^, reports of the translocation of MPs across the fish intestine must be viewed with caution, since the mechanisms of the passage of the plastic material outside of the fish GIT are not yet determined. Two main routes of translocation have been suggested: transcellular and paracellular^18,103^. The transcellular route involves absorption through the microvillous border to the blood^93,103^, while the paracellular route occurs through the tight junctions between the cells into the blood^104^. In mammals, transcellular uptake occurs mostly via M cells in Peyer’s patches and gut-associated lymphoid tissue (GALT)^105,106^. Fish do not have an organized GALT but instead have lymphoid cells scattered throughout the epithelium and lamina propria and occasional macrophages. Until recently, it was believed that fish lacked M cells^107^. However, recent studies in salmonids^108^ and *D. rerio*^109^ identified specialized enterocytes with M cell-like activity in the posterior part of the mid-intestine. In fish, these parts of the mid-intestine are the major sites for the uptake of macromolecules and transfer to closely associated intra-epithelial macrophages^109^. In *D. rerio,* these cells are identified by the presence of large, supra-nuclear vacuoles^109^, and it has been suggested that these vacuolated cells deliver luminal contents to scattered immune cells present underneath the epithelial layer^110^*. *However, phagocytic activity is not limited to these cells and was also found in regular enterocytes^109^. The paracellular passage of solid particles through gaps between the enterocytes into the circulatory system has been suggested as the most likely route for MPs, owing to the size range they cover^18^. In cases of severe inflammation and erosion, the passage of particles through the damaged tissue appears to be facilitated^104,111^.

MPs could enter the circulatory system by either of these routes and reach the sinusoids through the endothelial fenestrae and the space of Disse. From here, uptake could take place across the basal membrane of the hepatocytes^112^. The fenestrae vary in size, depending on physiological and pathological conditions, controlling what goes in to or out of the space of Disse and what the hepatocytes are exposed to^113^. Latex beads of 1 µm and 100 nm were observed within the hepatic sinusoids in both juvenile and adult *D. rerio*^113^.

To the best of our knowledge, our study is the first to confirm the internalization of MP particles in the liver, thus validating the translocation of MP particles. In the present study, the translocation of particles was limited to the liver, as no other organs or tissues showed the presence of MPs, despite other authors having described translocation to the muscle^37,47^.

#### Histopathological changes

The digestive tract of fishes is an extension of the external environment, acting as a critical interface between the internal and external environments^49^ and hence being considered a major route of exposure to MPs. For that reason, intestinal samples were assessed following the guidelines described by Saraiva et al.^62^.

Our observations revealed the absence of significant lesions in *D. rerio* from both treatment groups after sub-chronic dietary exposure. Similar results were reported in *S. aurata*^47^ and *Oncorhynchus mykiss*^58^ fed different types of pristine MPs (﻿0.1 g/kg bodyweight/day) for 45 days and exposed to both pristine and environmentally deployed PS MPs (﻿10 mg/fish/day) for 4 weeks. Similarly, *Barbodes gonionotus* exposed to PVC fragments (0.2, 0.5 and 1.0 mg/L) for 96 h did not present evident tissue damage^114^.

Nonetheless, after an exhaustive review of the scientific literature published on the effects of pristine MPs on fish intestines, we realized that the majority of publications present distinct results. Previous intestinal histological changes reported in fish exposed to pristine MPs included cilia defects in adult *D. rerio*^79^. As ciliated cells are found in only the intestinal epithelia of lampreys, chondrosteans and dipnoids as well as in early life stages in some teleosts^115^, they are not present in the intestinal mucosa of most fish species (e.g., *D. rerio)*. In most cases, fish have brush border microvilli instead^115^, lined by layers of water and mucus^49^.

Regressive changes, such as erosion and/or ulceration^78^ of the mucosa, have been reported under the following synonyms: epithelial and villi damage^56^, cracking of villi, splitting of the enterocytes^57^ or breakage of the epithelium^78^. However, such observations must be taken with caution, as erosion and, especially, ulceration are often accompanied by necrosis or haemorrhaging of the mucosa^116^; these changes are not evident in the photomicrographs offered by any of the aforementioned authors. The detachment of the epithelium from the lamina propria has allegedly been observed^55,78,117,118^. However, the photomicrographs given to support these observations show a separation between the mucosal epithelium and the lamina propria, which is a common preparation artefact. Other findings, described as a shortening and fusion of the mucosal folds, have also been documented^55^. Even though persistent toxic damage to the intestinal mucosa and chronic inflammation can produce morphological changes in intestinal folds, such as atrophy and the fusion of adjacent folds, the illusory appearance of these lesions in transverse sections can also be caused by plane-of-section artefacts^64^. This appears to be the case in the aforementioned study^55^. Beheading of villi^55^ has also been reported. Is important to bear in mind that examination of the intestine may be problematic since artefacts due to autolysis occur quickly^119^. The autolysis of the tips of mucosal folds is a common artefactual finding and often occurs when whole fish are fixed^72^. Thus, care should be taken when ascribing pathological significance to autolytic changes^72^.

Vacuolation of enterocytes has been described^55,79,117^. However, when assessing the vacuolation of the enterocytes in some fish species (i.e., *D. rerio*), the presence of specialized enterocytes in the posterior segment of the mid-intestine with prominent supranuclear vacuoles has to be taken into account^120^. A previous study^79^ has characterized the vacuolation of what looks like a normal enterocyte in the posterior segment of the mid-intestine as a pathological change.

Regarding progressive changes, in our study, the number and size of goblet cells did not show significant differences among the test groups. Likewise, a study conducted on *Onchorhyncus mykiss* fed PS MPs showed no significant changes in the numbers of goblet cells observed^58^. Although prior studies^95,118^ have mentioned mucous hypersecretion**,** none of the photomicrographs provided showed a significant presence of mucous secretion. In both cases, the authors pointed out to the goblet cells, but mucous hypersecretion was not evident. Thickness of the mucous layer is also known to change by region, being generally greater in the distal sections of the intestine^121^. Hyperplasia^55,117^ and hypertrophy of the goblet cells^78^ have also been reported. Mucous cell hyperplasia is generally associated with sources of persistent irritation, such as parasitism^122^ and diet^119^. On the other hand, a decrease in the mucus volume^79^ and a reduction in the number of goblet cells^118^ have been reported. Goblet cells are known to vary along the intestinal length^121^, and for many species of teleosts, the posterior intestine contains the highest concentration of goblet cells^123^. Fasting has also been shown to reduce the mucosal mass of the intestine^124^. Another reported finding was hyperplasia of the rodlet cells^55^. However, no evidence of hyperplasia was given by the photomicrographs available, as rodlet cells can be found in low numbers in healthy tissues. Additionally, care should be taken when ascribing pathological significance to the presence or abundance of these cells because no consistent relation has been established between the numbers of rodlet cells and disease^64^.

In the present study, inflammation was not observed. Scattered lymphocytes were present in the lamina propria; however, these were considered part of the normal lymphoid tissue. Inflammatory changes^56,78^ as well as the presence of neutrophils in the intestinal mucosa^118^ and mast cells at the base of the epithelium^79^ were observed by other authors. However, these observations are not discernible in the photomicrographs available. The presence of a few leukocytes per se does not necessarily translate to a pathological finding and/or inflammation. In healthy fish, there is a resident population of leukocytes, such as mast cells/eosinophilic granule cells (EGCs), and lymphocytes scattered in the lamina propria^62^.

Owing to its large blood supply and marked metabolic capacity, the liver is a target organ for toxicants^112^, while also providing pertinent information about general health and revealing the existence of subclinical background diseases^65^. In the present study, liver samples were evaluated separately, according to fish gender, following the protocol proposed by Bernet et al*.*^63^ Specific sex-related differences are characteristic in adult *D. rerio*. In reproductively active, adult oviparous females, an upregulated synthesis of the egg yolk protein vitellogenin often causes the hepatocyte cytoplasm to have a mottled, basophilic appearance, with collapsed sinusoids, owing to the hepatocyte enlargement^64,124^. In contrast, livers from reproductively active males have round eosinophilic hepatocytes with clear vacuoles containing slightly flocculent material and minimal displacement of the nucleus, consistent with glycogen^124^.

Overall, no circulatory, proliferative, inflammatory or neoplastic changes were noted in our study. After a thorough review of the scientific literature on the effects of pristine MPs on fish livers, we observed that our findings were contrary to those of previous studies, suggesting the occurrence of several changes as a consequence of MP exposure.

Circulatory changes, namely congestion and hyperemia^78,117^ and haemorrhaging^125^, have been reported. In a particular case^78^, the finding described as congestion is likely intravascular eosinophilic proteinaceous fluid. Similar findings were documented by van der Ven et al*.*^126^, who identified these changes as an accumulation of vitellogenin in vessels. However, the gender of the animals used by Jabeen et al*.*^78^ was not disclosed, and further conclusions cannot be drawn. Overall, when assessing liver congestion and dilated sinusoids, it is important to bear in mind the degree to which the fish was exsanguinated at sacrifice and the amount of care taken to not manually squeeze the liver sample at necropsy^64^. Liver haemorrhaging^125^ seems to be characterized by a small number of erythrocytes within the sinusoids, which were likely severed during microtomy. Reports of congestion or dilated sinusoids often are artefacts of tissue collection, preservation or processing^65^.

Regarding the regressive changes in our study, 22.2% (4/18) of the females presented minimal to mild vacuolation (score: 1–2), while to 27.8% (5/18) presented moderate vacuolation (score: 4). Additionally, 27.8% (5/18) of the males displayed both minimal and mild vacuolation (score: 1–2). A reduction in vacuolation in both sexes appeared to be time-dependent. Glycogen depletion was similarly described in *Oryzias latipes* exposed to pristine MPs^127^. The loss of hepatocellular vacuolation is a common response of fish livers to toxicity^128^. Furthermore, it is a non-specific finding that can occur as a direct effect of intoxication or secondary to decreased body condition caused by inanition, stress or concurrent disease^64,124^. In our case, vacuolation was not significantly correlated with fish weight, and concurrent diseases were not identified. We believed that the loss of hepatocellular vacuolation might have been caused by stress or even by prolonged exposure to MPs. Paradoxically, toxic exposure can also result in the accumulation of lipids or glycogen in the liver^124^.

Increased hepatocellular vacuolation^117^ and vacuolar swelling^129^ in fish exposed to pristine MPs have also been reported by other authors. However, care must be taken before considering increased hepatocellular vacuolation a pathological change, as it can also be the result of overfeeding an excessively energy-rich diet or lipid peroxidation^124^. It has been suggested that captive marine teleosts may be particularly predisposed to hepatic lipidosis, as observed in *D. labrax* used in a study with MPs^117^, owing to a reduced capacity for hepatocyte peroxisome proliferation coupled with the feeding of artificial diets with high proportions of mono-unsaturated fatty acids^124^. Lipid peroxidation in fish may also be toxicant-induced^65,124^. In another study^48^, lipid droplets in hepatocytes were reported. However, further histochemical techniques were not performed to confirm the lipid origin of the vacuolation. Additionally, due to artefacts in the control photomicrograph, it is difficult to establish a comparison between the control and test groups to identify a possible increase in hepatocellular vacuolation. Apart from lipid and glycogen vacuolation, there are other potential causes of hepatocellular enlargement, such as vacuolar swelling of the endoplasmic reticulum cisternae (hydropic degeneration)^124^. Vacuolar swelling and hydropic degeneration were allegedly observed in another study^78^. When comparing the control and test group photomicrographs, it can be observed that cells meant to illustrate hydropic degeneration are also present in the control group photomicrograph. As the magnification differs between the images, there is an illusion of larger vacuoles in the test group photomicrograph. The same applies for the reported vacuolar swelling.

Necrosis was also reportedly observed in the livers of fish exposed to MPs^48,117,125^. In one study^125^, necrosis was likely due to handling trauma. Clear spaces without tissue are more likely to be artefacts resulting from focal trauma during tissue collection. In other experiments^48,125^, there was no evidence of necrosis. Findings classified as hepatocellular necrosis or apoptosis should display cytoplasmic hypereosinophilia with or without condensation; irregular or rounded cytoplasmic margins; nuclear changes, such as pyknosis, karyorrhexis or karyolysis; phagocytosis of necrotic cells or apoptotic bodies; and in the case of necrosis, a potential inflammatory response^65^. Damaged and aggregated nuclei were documented^129^. However, these are likely a proliferation of macrophage aggregates. Starvation, ageing, infectious diseases and toxins are all likely to cause proliferation of macrophage aggregates^124^.

Among the progressive changes, alterations in the hepatocyte morphology and hypertrophy^117^ were reported. Often, when increased vacuolation results in cytoplasmic enlargement, there is confusion with hepatocellular hypertrophy^65^. However, hepatocellular hypertrophy should be reserved for describing non-vacuolated cells that are enlarged as a consequence of metabolic enzyme induction, which results in an upregulation of organelles^65^. In addition, whether due to physiological or toxicological causes, hepatocyte hypertrophy is often accompanied by basophilia^124^.

Inflammation was also described^48,78^. In the first study^48^, signs of inflammation were not evident in any of the provided photomicrographs. In turn, the photomicrographs provided in the second study^78^ appeared to identify a tubular structure, likely part of the biliary system^130^ or, less likely, a vascular structure of a normal hepatic stroma. Even though there might be a few leukocytes in the figure provided^78^, rare leukocytes do not necessarily mean inflammation. When assessing inflammation in the liver, one has to take into account that haemopoietic tissue may be present in the periportal areas of the liver in some fish species^131^ and that an integral component of inflammation is the infiltration of non-resident leukocytes into the affected site^65^.

The specific architectural design of fish livers, having only the basal and basolateral aspects of hepatocytes directly exposed to the sinusoidal perfusion, hinders the uptake of chemicals by fish hepatocytes^124^. This and the lower perfusion rate might help to explain the relative tolerance of fishes to MPs.

Our results show that *D. rerio* individuals recognize plastic particles as inedible materials but ingest them either when they are mixed with food or fish oil or accidentally when exposed to relatively small plastic particles (1–5 µm). Ingested small plastic microbeads (1–5 µm) and medium PE microfragments (120–255 µm) and fibres (average width and length of 13.67 µm and 1.5 mm, respectively) are unlikely to accumulate in the digestive tract of *D. rerio* after one or multiple meals, as MPs were almost completely evacuated after 24 h and only a few particles remained in the digestive tract after sub-chronic ingestion. No mortalities or significant effects on body condition were identified after 45 days of feeding with MPs. However, the fish fed medium-sized, irregular PE MPs showed anorexia and lethargy. The ingestion of particles has been reported to cause physical blockage of the intestine, causing a false sense of satiety and interfering with feeding^23,34^. In the present case, the relatively large particles are thought to have impaired feeding.

To the best of our knowledge, this is the first study to fully demonstrate the uptake and translocation of plastic microbeads to the liver using confocal microscopy. However, the exact route through which MPs reach the liver is still unknown, and future studies are necessary to determine the mechanisms that allow the uptake and translocation of MPs.

Following sub-chronic dietary exposure to pristine MPs, *D. rerio* did not show any histological lesions in the observed organs. Our results are in contrast to the majority of the scientific literature on the effects of MPs in fish. The differences may be influenced by several elements, such as the species, age, sex, reproductive status of the fish, environment, tested concentrations, size, type, surface chemistry and hydrophobicity of MPs, feeding routine, exposure route, exposure time, number of animals and replicates per treatment group, specimen collection and preparation methods^65^. However, inaccuracy in the interpretation of the histopathological findings may be the main cause for the disparity observed in the results regarding the effects of pristine MPs on fish. A letter^54^ written by several veterinary pathologists has highlighted concerns about the recurring problem of inaccurate histopathological data, which is increasingly observed in scientific publications**.** This situation is especially alarming in cases in which the study conclusions depend heavily on the histopathological results. In addition, such observations will persist in the literature and spawn further misguided research, which is particularly problematic for students and researchers working in fish pathology, expecting to find reliable sources of information in these same publications.

Although pristine MPs per se do not appear to produce imminent damage, most plastics produced are not made entirely of plastic polymers. During the manufacture of plastics, endogenous chemical additives are incorporated into them^18^. MPs are also very efficient in adsorbing persistent organic pollutants already present in water^23^. Therefore, further research is needed to properly identify the effects MPs and their associated contaminants may have on animal health and, consequently, public health.

## Supplementary information

Supplementary Figures.

Supplementary Tables.

## References

[1] Wright SL, Thompson RC, Galloway TS (2013). The physical impacts of microplastics on marine organisms: a review. Environ. Pollut..

[2] Toussaint B (2019). Review of micro- and nanoplastic contamination in the food chain. Food Addit. Contam. Part A Chem. Anal. Control Expo Risk Assess..

[3] Thompson RC, Moore CJ, Saal FSV, Swan SH (2009). Plastics, the environment and human health: Current consensus and future trends. Philos. Trans. R. Soc. B..

[4] Carpenter EJ, Smith KL (1972). Plastics on the Sargasso sea surface. Science.

[5] Reisser J (2013). Marine plastic pollution in waters around Australia: characteristics, concentrations, and pathways. PLoS ONE.

[6] Eriksen M (2013). Microplastic pollution in the surface waters of the Laurentian Great Lakes. Mar. Pollut. Bull..

[7] Faure F, Demars C, Wieser O, Kunz M, de Alencastro LF (2015). Plastic pollution in Swiss surface waters: nature and concentrations, interaction with pollutants. Environ. Chem..

[8] Lusher AL, Tirelli V, O’Connor I, Officer R (2015). Microplastics in Arctic polar waters: the first reported values of particles in surface and sub-surface samples. Sci. Rep..

[9] Lebreton L (2018). Evidence that the Great Pacific Garbage Patch is rapidly accumulating plastic. Sci. Rep..

[10] von Moos N, Burkhardt-Holm P, Köhler A (2012). Uptake and effects of microplastics on cells and tissue of the blue mussel *Mytilus edulis* L. after an experimental exposure. Environ. Sci. Technol..

[11] Browne MA, Galloway T, Thompson R (2007). Microplastic—an emerging contaminant of potential concern. Integr. Environ. Assess. Manag..

[12] Crawford CB, Quinn B (2017). Microplastic Pollutants. Ch. 4.

[13] Kemikalieinspektionen (KEMI). *Mikroplast i kosmetiska produkter och andra kemiska produkter—Rapport från ett regeringsuppdrag*. Kemi Report 2/18 (2018). Available at: https://www.kemi.se/en/global/rapporter/2018/rapport-2-18-mikroplast-i-kosmetiska-produkter-och-andra-kemiska-produkter.pdf (Accessed 1st June 2020).

[14] Dris R (2015). Microplastic contamination in an urban area: a case study in Greater Paris. Environ. Chem..

[15] Fischer EK, Paglialonga L, Czech E, Tamminga M (2016). Microplastic pollution in lakes and lake shoreline sediments—a case study on Lake Bolsena and Lake Chiusi (central Italy). Environ. Pollut..

[16] Jabeen K (2016). Microplastics and mesoplastics in fish from coastal and fresh waters of China. Environ. Pollut..

[17] Su L (2016). Microplastics in Taihu Lake, China. Environ. Pollut..

[18] Wright SL, Kelly FJ (2017). Plastic and human health: a micro issue?. Environ. Sci. Technol..

[19] Free CM (2014). High-levels of microplastic pollution in a large, remote, mountain lake. Mar. Pollut. Bull..

[20] Ballent A, Corcoran PL, Madden O, Helm PA, Longstaffe FJ (2016). Sources and sinks of microplastics in Canadian Lake Ontario nearshore, tributary and beach sediments. Mar. Pollut. Bull..

[21] Mani T, Hauk A, Wal U, Burkhardt-Holm P (2016). Microplastics profile along the Rhine River. Sci. Rep..

[22] Critchell K, Hoogenboom MO (2018). Effects of microplastic exposure on the body condition and behaviour of planktivorous reef fish (*Acanthochromis polyacanthus*). PLoS ONE.

[23] Jovanović B (2017). Ingestion of microplastics by fish and its potential consequences from a physical perspective. Integr. Environ. Assess. Manag..

[24] Batel A, Linti F, Scherer M, Erdinger L, Braunbeck T (2016). Transfer of benzo[a]pyrene from microplastics to *Artemia nauplii* and further to zebrafish via a trophic food web experiment: CYP1A induction and visual tracking of persistent organic pollutants. Environ. Toxicol. Chem..

[25] Frias JPGL, Otero V, Sobral P (2014). Evidence of microplastics in samples of zooplankton from Portuguese coastal waters. Mar. Environ. Res..

[26] Van Cauwenberghe L, Claessens M, Vandegehuchte MB, Janssen CR (2015). Microplastics are taken up by mussels (*Mytilus edulis*) and lugworms (*Arenicola marina*) living in natural habitats. Environ. Pollut..

[27] Nobre CR (2015). Assessment of microplastic toxicity to embryonic development of the sea urchin *Lytechinus variegatus* (Echinodermata: Echinoidea). Mar. Pollut. Bull..

[28] Green DS, Boots B, Sigwart J, Jiang S, Rocha C (2016). Effects of conventional and biodegradable microplastics on a marine ecosystem engineer (*Arenicola marina*) and sediment nutrient cycling. Environ. Pollut..

[29] Hu L (2016). Uptake, accumulation and elimination of polystyrene microspheres in tadpoles of *Xenopus tropicalis*. Chemosphere.

[30] Duncan EM (2019). Microplastic ingestion ubiquitous in marine turtles. Glob. Change Biol..

[31] Besseling E (2015). Microplastic in a macro filter feeder: humpback whale *Megaptera novaeangliae*. Mar. Pollut. Bull..

[32] Amélineau F (2016). Microplastic pollution in the Greenland Sea: background levels and selective contamination of planktivorous diving seabirds. Environ. Pollut..

[33] Provencher JF, Vermaire JC, Avery-Gomm S, Braune BM, Mallory ML (2018). Garbage in guano? Microplastic debris found in faecal precursors of seabirds known to ingest plastics. Sci. Total Environ..

[34] Neves D, Sobral P, Ferreira JL, Pereira T (2015). Ingestion of microplastics by commercial fish off the Portuguese coast. Mar. Pollut. Bull..

[35] Naidoo T, Smit AJ, Glassom D (2016). Plastic ingestion by estuarine mullet *Mugil cephalus* (Mugilidae) in an urban harbour, KwaZulu-Natal, South Africa. Afr. J. Mar. Sci..

[36] Tanaka K, Takada H (2016). Microplastic fragments and microbeads in digestive tracts of planktivorous fish from urban coastal waters. Sci. Rep..

[37] Abbasi S (2018). Microplastics in different tissues of fish and prawn from the Musa Estuary, Persian Gulf. Chemosphere.

[38] Herrera A (2019). Microplastic ingestion by Atlantic chub mackerel *(Scomber colias*) in the Canary Islands coast. Mar. Pollut. Bull..

[39] Boerger CM, Lattin GL, Moore SL, Moore CJ (2010). Plastic ingestion by planktivorous fishes in the North Pacific Central Gyre. Mar. Pollut. Bull..

[40] Nadal MA, Alomar C, Deudero S (2016). High levels of microplastic ingestion by the semipelagic fish bogue *Boops boops* (L.) around the Balearic Islands. Environ. Pollut..

[41] Güven O, Gökdağ K, Jovanović B, Kıdeyş AE (2017). Microplastic litter composition of the Turkish territorial waters of the Mediterranean Sea, and its occurrence in the gastrointestinal tract of fish. Environ. Pollut..

[42] Silva-Cavalcanti JS, Silva JDB, de França EJ, de Araújo MCB, Gusmão F (2017). Microplastics ingestion by a common tropical freshwater fishing resource. Environ. Pollut..

[43] Barboza LGA (2019). Microplastics in wild fish from North East Atlantic Ocean and its potential for causing neurotoxic effects, lipid oxidative damage, and human health risks associated with ingestion exposure. Sci. Total Environ..

[44] Su L (2019). The occurrence of microplastic in specific organs in commercially caught fishes from coast and estuary area of east China. J. Hazard. Mater..

[45] Grigorakis S, Mason SA, Drouillard KG (2017). Determination of the gut retention of plastic microbeads and microfibers in goldfish *(Carassius auratus)*. Chemosphere.

[46] Ory NC, Gallardo C, Lenz M, Thiel M (2018). Capture, swallowing, and egestion of microplastics by a planktivorous juvenile fish. Environ. Pollut..

[47] Jovanović B, Gökda K, Güven O, Emre Y, Whitley EM (2018). Virgin microplastics are not causing imminent harm to fish after dietary exposure. Mar. Pollut. Bull..

[48] Lu Y (2016). Uptake and accumulation of polystyrene microplastics in zebrafish (*Danio rerio*) and toxic effects in liver. Environ. Sci. Technol..

[49] Bakke AM, Glover C, Krogdahl A, Grosell M, Farrel AP, Brauner CJ (2011). Feeding, digestion and absorption of nutrients. Fish Physiology: The Multifunctional Gut of Fish.

[50] Avio CG, Gorbi S, Regoli F (2015). Experimental development of a new protocol for extraction and characterization of microplastics in fish tissues: first observations in commercial species from Adriatic Sea. Mar. Environ. Res..

[51] Collard F (2017). Microplastics in livers of European anchovies (*Engraulis encrasicolus*, L.). Environ. Pollut..

[52] Ding J, Zhang S, Razanajatovo RM, Zou H, Zhu W (2018). Accumulation, tissue distribution, and biochemical effects of polystyrene microplastics in the freshwater fish red tilapia (*Oreochromis niloticus*). Environ. Pollut..

[53] Schür C (2019). When fluorescence is not a particle: the tissue translocation of microplastics in *Daphnia magna* seems an artifact. Environ. Toxicol. Chem..

[54] Baumann L, Schmidt-Posthaus H, Segner H, Wolf JC (2016). Comment on “uptake and accumulation of polystyrene microplastics in zebrafish (*Danio rerio*) and toxic effects in liver”. Environ. Sci. Technol..

[55] Pedà C (2016). Intestinal alterations in European sea bass *Dicentrarchus labrax* (Linnaeus, 1758) exposed to microplastics: preliminary results. Environ. Pollut..

[56] Qiao R (2019). Microplastics induce intestinal inflammation, oxidative stress, and disorders of metabolome and microbiome in zebrafish. Sci. Total Environ..

[57] Lei L (2018). Microplastic particles cause intestinal damage and other adverse effects in zebrafish (*Danio rerio*) and nematode *Caenorhabditis elegans*. Sci. Total Environ..

[58] Ašmonait G, Sundh H, Asker N, Almroth BC (2018). Rainbow trout maintain intestinal transport and barrier functions following exposure to polystyrene microplastics. Environ. Sci. Technol..

[59] Deng Y, Zhang Y, Lemos B, Ren H (2017). Tissue accumulation of microplastics in mice and biomarker responses suggest widespread health risks of exposure. Sci. Rep..

[60] Braeuning A (2019). Uptake of microplastics and related health effects: a critical discussion of Deng et al., Scientific reports 7:46687, 2017. Arch. Toxicol..

[61] Bancroft JD, Layton C, Suvarna SK (2013). Bancroft's Theory and Practice of Histological Techniques.

[62] Saraiva A, Costa J, Serrão J, Cruz C, Eiras JC (2015). A histology-based fish health assessment of farmed seabass (*Dicentrarchus labrax* L.). Aquaculture.

[63] Bernet D, Schmidt H, Meier W, Burkhardt-Holm P, Wahli T (1999). Histopathology in fish: proposal for a protocol to assess aquatic pollution. J. Fish Dis..

[64] Wolf JC (2015). Nonlesions, misdiagnoses, missed diagnoses, and other interpretive challenges in fish histopathology studies: a guide for investigators, authors, reviewers, and readers. Toxicol. Pathol..

[65] Wolf JC, Wheeler JR (2018). A critical review of histopathological findings associated with endocrine and non-endocrine hepatic toxicity in fish models. Aquat. Toxicol..

[66] Lusher, A. L., Hollman, P. C. H. & Mendoza-Hill, J. *Microplastics in fisheries and aquaculture: status of knowledge on their occurrence and implications for aquatic organisms and food safety*. FAO fisheries and aquaculture technical *paper* No. 615. (2017) Available at: https://www.fao.org/documents/card/es/c/59bfa1fc-0875-4216-bd33-55b6003cfad8/ (Accessed: 19th December 2019).

[67] Anbumani S, Kakkar P (2018). Ecotoxicological effects of microplastics on biota: a review. Environ. Sci. Pollut. Res..

[68] Zhang K (2016). Microplastic pollution of lakeshore sediments from remote lakes in Tibet plateau, China. Environ. Pollut..

[69] Castañeda RA, Avlijas S, Simard MA, Ricciardi A (2014). Microplastic pollution in St. Lawrence River sediments. Can. J. Fish. Aquat. Sci..

[70] McCormick AR (2016). Microplastic in surface waters of urban rivers: concentration, sources, and associated bacterial assemblages. Ecosphere.

[71] Nel HA, Dalu T, Wasserman RJ (2018). Sinks and sources : assessing microplastic abundance in river sediment and deposit feeders in an Austral temperate urban river system. Sci. Total Environ..

[72] Lima ARA, Costa MF, Barletta M (2014). Distribution patterns of microplastics within the plankton of a tropical estuary. Environ. Res..

[73] Sadri SS, Thompson RC (2014). On the quantity and composition of floating plastic debris entering and leaving the Tamar Estuary, Southwest England. Mar. Pollut. Bull..

[74] Yonkos LT, Friedel EA, Perez-Reyes AC, Ghosal S, Arthur CD (2014). Microplastics in four estuarine rivers in the Chesapeake Bay, U.S.A.. Environ. Sci. Technol..

[75] Cózar A (2015). Plastic accumulation in the Mediterranean sea. PLoS ONE.

[76] Desforges JPW, Galbraith M, Dangerfield N, Ross PS (2014). Widespread distribution of microplastics in subsurface seawater in the NE Pacific Ocean. Mar. Pollut. Bull..

[77] Obbard RW (2014). Global warming releases microplastic legacy frozen in Arctic Sea ice. Earths Future..

[78] Jabeen K (2018). Effects of virgin microplastics on goldfish (*Carassius auratus*). Chemosphere.

[79] Qiao R (2019). Accumulation of different shapes of microplastics initiates intestinal injury and gut microbiota dysbiosis in the gut of zebrafish. Chemosphere.

[80] Choi JS, Jung YJ, Hong NH, Hong SH, Park JW (2018). Toxicological effects of irregularly shaped and spherical microplastics in a marine teleost, the sheepshead minnow (*Cyprinodon variegatus*). Mar. Pollut. Bull..

[81] Kim SW, Chae Y, Kim D, An YJ (2019). Zebrafish can recognize microplastics as inedible materials: quantitative evidence of ingestion behaviour. Sci. Total Environ..

[82] Colton JB, Burns BR, Knapp FD (1974). Plastic particles in surface waters of the Northwestern Atlantic. Science.

[83] Dantas DV, Barletta M, Ferreira M (2012). The seasonal and spatial patterns of ingestion of polyfilament nylon fragments by estuarine drums (Sciaenidae). Environ. Sci. Pollut. Res. Int..

[84] Sanchez W, Bender C, Porcher JM (2014). Wild gudgeons (*Gobio gobio*) from French rivers are contaminated by microplastics: preliminary study and first evidence. Environ. Res..

[85] Biginagwa FJ, Mayoma BS, Shashoua Y, Syberg K, Khan FR (2016). First evidence of microplastics in the African Great Lakes: recovery from Lake Victoria Nile perch and Nile tilapia. J. Great Lakes Res..

[86] Pazos RS, Maiztegui T, Colautti DC, Paracampo AH, Gómez N (2017). Microplastics in gut contents of coastal freshwater fish from Río de la Plata estuary. Mar. Pollut. Bull..

[87] Bessa F (2018). Occurrence of microplastics in commercial fish from a natural estuarine environment. Mar. Pollut. Bull..

[88] de Sá LC, Luís LG, Guilhermino L (2015). Effects of microplastics on juveniles of the common goby (*Pomatoschistus microps*): confusion with prey, reduction of the predatory performance and efficiency, and possible influence of developmental conditions. Environ. Pollut..

[89] Savoca MS, Tyson CW, McGill M, Slager CJ (2017). Odours from marine plastic debris induce food search behaviours in a forage fish. Proc. R. Soc. B..

[90] Finney JL, Robertson GN, McGee CA, Smith FM, Croll RP (2006). Structure and autonomic innervation of the swim bladder in the zebrafish (*Danio rerio*). J. Comp. Neurol..

[91] Fabian B (1983). Persorption—the way of large sized corpuscle particles via the lymphatic system. Lymphology..

[92] Birchenough GMH, Johansson MEV, Gustafsson JK, Bergström JH, Hansson GC (2015). New developments in goblet cell mucus secretion and function. Mucosal. Immunol..

[93] Carr KE, Smyth SH, McCullough MT, Morris JF, Moyes SM (2012). Morphological aspects of interactions between microparticles and mammalian cells: intestinal uptake and onward movement. Prog. Histochem. Cytochem..

[94] Santos J, Jobling M (1991). Gastric emptying in cod, *Gadus morhua* L.: emptying and retention of indigestible solids. J. Fish Biol..

[95] Jin Y (2018). Polystyrene microplastics induce microbiota dysbiosis and inflammation in the gut of adult zebrafish. Environ. Pollut..

[96] Wen B (2018). Microplastics have a more profound impact than elevated temperatures on the predatory performance, digestion and energy metabolism of an Amazonian cichlid. Aquat. Toxicol..

[97] Espinosa C, Cuesta A, Esteban MA (2017). Effects of dietary polyvinylchloride microparticles on general health, immune status and expression of several genes related to stress in gilthead seabream (*Sparus aurata* L.). Fish Shellfish Immunol..

[98] Yin L, Chen B, Xia B, Shi X, Qu K (2018). Polystyrene microplastics alter the behavior, energy reserve and nutritional composition of marine jacopever (*Sebastes schlegelii*). J. Hazard. Mater..

[99] Astrofsky KM, Harper CM, Rogers AB, Fox JG (2002). Diagnostic techniques for clinical investigation of laboratory zebrafish. Lab. Anim..

[100] Kalueff AV (2013). Towards a comprehensive catalog of zebrafish behavior 1.0 and beyond. Zebrafish..

[101] Moore CJ, Lattin GL, Zellers AF (2011). Quantity and type of plastic debris flowing from two urban rivers to coastal waters and beaches of Southern California. J. Integr. Coast. Zone Manag..

[102] Andrady AL (2017). The plastic in microplastics: a review. Mar. Pollut. Bull..

[103] Bouwmeester H, Hollman PCH, Peters RJB (2015). Potential health impact of environmentally released micro- and nanoplastics in the human food production chain: experiences from nanotoxicology. Environ. Sci. Technol..

[104] Handy RD, Henry TB, Scown TM, Johnston BD, Tyler CR (2008). Manufactured nanoparticles: their uptake and effects on fish—a mechanistic analysis. Ecotoxicology.

[105] Hussain N, Jaitley V, Florence AT (2001). Recent advances in the understanding of uptake of microparticulates across the gastrointestinal lymphatics. Adv. Drug Deliv. Rev..

[106] Behrens I, Pena AIV, Alonso MJ, Kissel T (2002). Comparative uptake studies of bioadhesive and non-bioadhesive nanoparticles in human intestinal cell lines and rats—the effect of mucus on particle adsorption and transport. Pharm. Res..

[107] Jovanović B, Palić DŠ (2012). Immunotoxicology of non-functionalized engineered nanoparticles in aquatic organisms with special emphasis on fish—review of current knowledge, gap identification, and call for further research. Aquat. Toxicol..

[108] Fuglem B (2010). Antigen-sampling cells in the salmonid intestinal epithelium. Dev. Comp. Immunol..

[109] Løvmo SD (2017). Translocation of nanoparticles and *Mycobacterium marinum* across the intestinal epithelium in zebrafish and the role of the mucosal immune system. Dev. Comp. Immunol..

[110] Brugman S (2016). The zebrafish as a model to study intestinal inflammation. Dev. Comp. Immunol..

[111] Volkheimer G (1975). Hematogenous dissemination of ingested polyvinyl chloride particles. Ann. N. Y. Acad. Sci..

[112] Hinton DE, Segner H, Braunbeck T, Schlenk D, Benson WH (2001). Toxic responses of the liver. Target Organ Toxicity in Marine and Freshwater Teleosts: Volume I. Ch. 4.

[113] Cheng, B. D. Structure–function properties of the gastrodigestive and hepatic systems of zebrafish (*Danio rerio.* (2018) Available at: https://ses.library.usyd.edu.au/handle/2123/19797 (Accessed: 3rd February 2020).

[114] Romano N, Ashikin M, Teh JC, Syukri F, Karami A (2018). Effects of pristine polyvinyl chloride fragments on whole body histology and protease activity in silver barb *Barbodes gonionotus* fry. Environ. Pollut..

[115] Wilson JM, Castro LFC, Grosell M, Farrel AP, Brauner CJ (2011). Morphological diversity of the gastrointestinal tract in fishes. Fish Physiology: The Multifunctional Gut of FishCh1.

[116] Wallig MA, Janovitz EB, Wallig MA, Haschek WM, Rousseaux CG, Bolon B, Mahler BW (2018). Morphologic manifestations of toxic cell injury. Fundamentals of Toxicologic Pathology. Ch. 5.

[117] Espinosa C, Esteban MA, Cuesta A (2019). Dietary administration of PVC and PE microplastics produces histological damage, oxidative stress and immunoregulation in European sea bass (*Dicentrarchus labrax* L.). Fish Shellfish Immunol..

[118] Limonta G (2019). Microplastics induce transcriptional changes, immune response and behavioral alterations in adult zebrafish. Sci. Rep..

[119] Lumsden JS, Ferguson HW (2006). Gastrointestinal tract, swimbladder, pancreas and peritoneum. Systemic Pathology of Fish. Ch. 7.

[120] Wallace KN, Akhter S, Smith EM, Lorent K, Pack M (2005). Intestinal growth and differentiation in zebrafish. Mech. Dev..

[121] Kleinow KM, Nichols JW, Hayton WL, McKim JM, Barron MG, Giulio RTD, Hinton DE (2008). Toxicokinetics in fishes. The Toxicology of Fishes.

[122] Nickol B, Woo PTK (2006). Phylum acanthocephala. Fish Diseases and Disorders Volume 1. Ch. 13.

[123] Kleinow KM, James MO, Schlenk D, Benson WH (2001). Response of the Teleost gastrointestinal system to xenobiotics. Target Organ Toxicity in Marine and Freshwater Teleosts Volume I. Ch. 5.

[124] Wolf JC, Wolfe MJ (2005). A brief overview of nonneoplastic hepatic toxicity in fish. Toxicol. Pathol..

[125] Karami A, Romano N, Galloway T, Hamzah H (2016). Virgin microplastics cause toxicity and modulate the impacts of phenanthrene on biomarker responses in African catfish (*Clarias gariepinus*). Environ. Res..

[126] Van Der Ven LTM (2003). Vitellogenin expression in zebrafish *Danio rerio*: evaluation by histochemistry, immunohistochemistry, and in situ mRNA hybridisation. Aquat. Toxicol..

[127] Rochman CM, Hoh E, Kurobe T, Teh SJ (2013). Ingested plastic transfers hazardous chemicals to fish and induces hepatic stress. Sci. Rep..

[128] Ferguson HW (1989). Systemic Pathology of Fish: A Text and Atlas of Comparative Tissue Responses in Diseases of Teleosts. Ch. 8.

[129] Chae Y, Kim D, Kim SW, An YJ (2018). Trophic transfer and individual impact of nano-sized polystyrene in a four-species freshwater food chain. Sci. Rep..

[130] Vliegenthart AD, Tucker CS, Del Pozo J, Dear JW (2014). Zebrafish as model organisms for studying drug-induced liver injury. Br. J. Clin. Pharmacol..

[131] Roberts RJ, Robers RJ (2012). The anatomy and physiology of teleosts. Fish Pathology. Ch. 2.

