# Supplementary material for: An end to the controversy over the microscopic detection and effects of pristine microplastics in fish organs
Source: Sci Rep. 2020 Jul 24;10:12434. doi: 10.1038/s41598-020-69062-3 (PMC7382502; doi:10.1038/s41598-020-69062-3)
Supplement: Supplementary file 2 — Supplementary Tables. [file 41598_2020_69062_MOESM2_ESM.pdf]

***An end to the controversy over the microscopic detection and effects of pristine microplastics in fish organs*.**

De Sales-Ribeiro, Carolina1*; Brito Casillas, Yeray2; Fernandez, Antonio1; Caballero, María José1

1 Division of Veterinary Histology and Pathology, Institute for Animal Health and Food Safety (IUSA), Veterinary School, University of Las Palmas de Gran Canaria, Arucas, 35413, Spain
2 Research Institute in Biomedical and Health Sciences (IUIBS), University of Las Palmas de Gran Canaria, Las Palmas de Gran Canaria, 35016, Spain

Supplementary Tables

Supplementary Table S1. Histopathological assessment tools for intestine.

				Circulatory Disturbances (rp1)							Regressive changes (rp2)																													
											Epithelium																		*Lamina Propria*											
	D. rerio	Test Group	Sex	Haemorrhage / hyperaemia / aneurysm			Intercelular oedema			I org rp	Architectural and strucutral alterations			Plasma alterations			Deposits			Nuclear alterations			Atrophy			Necrosis			Architectural and strucutral alterations			Plasma alterations			Deposits			Nuclear alterations		
				w	a	a x w	w	a	a x w		w	a	a x w	w	a	a x w	w	a	a x w	w	a	a x w	w	a	a x w	w	a	a x w	w	a	a x w	w	a	a x w	w	a	a x w	w	a	a x w
T0	1	CONTROL	F	1	0	0	1	0	0	0	1	0	0	1	0	0	1	0	0	2	0	0	2	0	0	3	0	0	1	0	0	1	0	0	1	0	0	2	0	0
	2		F	1	0	0	1	0	0	0	1	0	0	1	0	0	1	0	0	2	0	0	2	0	0	3	0	0	1	0	0	1	0	0	1	0	0	2	0	0
	3		M	1	0	0	1	0	0	0	1	0	0	1	0	0	1	0	0	2	0	0	2	0	0	3	0	0	1	0	0	1	0	0	1	0	0	2	0	0
	4		M	1	0	0	1	0	0	0	1	0	0	1	0	0	1	0	0	2	0	0	2	0	0	3	0	0	1	0	0	1	0	0	1	0	0	2	0	0
T30	1	FLUORESCENT	F	1	0	0	1	0	0	0	1	0	0	1	0	0	1	0	0	2	0	0	2	0	0	3	0	0	1	0	0	1	0	0	1	0	0	2	0	0
	2		F	1	0	0	1	0	0	0	1	0	0	1	0	0	1	0	0	2	0	0	2	0	0	3	0	0	1	0	0	1	0	0	1	0	0	2	0	0
	3		F	1	0	0	1	0	0	0	1	0	0	1	0	0	1	0	0	2	0	0	2	0	0	3	0	0	1	0	0	1	0	0	1	0	0	2	0	0
	4		M	1	0	0	1	0	0	0	1	0	0	1	0	0	1	0	0	2	0	0	2	0	0	3	0	0	1	0	0	1	0	0	1	0	0	2	0	0
	5		M	1	0	0	1	0	0	0	1	0	0	1	0	0	1	0	0	2	0	0	2	0	0	3	0	0	1	0	0	1	0	0	1	0	0	2	0	0
	6		M	1	0	0	1	0	0	0	1	0	0	1	0	0	1	0	0	2	0	0	2	0	0	3	0	0	1	0	0	1	0	0	1	0	0	2	0	0
	1	COSMETIC	F	1	0	0	1	0	0	0	1	0	0	1	0	0	1	0	0	2	0	0	2	0	0	3	0	0	1	0	0	1	0	0	1	0	0	2	0	0
	2		F	1	0	0	1	0	0	0	1	0	0	1	0	0	1	0	0	2	0	0	2	0	0	3	0	0	1	0	0	1	0	0	1	0	0	2	0	0
	3		F	1	0	0	1	0	0	0	1	0	0	1	0	0	1	0	0	2	0	0	2	0	0	3	0	0	1	0	0	1	0	0	1	0	0	2	0	0
	4		M	1	0	0	1	0	0	0	1	0	0	1	0	0	1	0	0	2	0	0	2	0	0	3	0	0	1	0	0	1	0	0	1	0	0	2	0	0
	5		M	1	0	0	1	0	0	0	1	0	0	1	0	0	1	0	0	2	0	0	2	0	0	3	0	0	1	0	0	1	0	0	1	0	0	2	0	0
	6		M	1	0	0	1	0	0	0	1	0	0	1	0	0	1	0	0	2	0	0	2	0	0	3	0	0	1	0	0	1	0	0	1	0	0	2	0	0
	1	CONTROL	F	1	0	0	1	0	0	0	1	0	0	1	0	0	1	0	0	2	0	0	2	0	0	3	0	0	1	0	0	1	0	0	1	0	0	2	0	0
	2		F	1	0	0	1	0	0	0	1	0	0	1	0	0	1	0	0	2	0	0	2	0	0	3	0	0	1	0	0	1	0	0	1	0	0	2	0	0
	3		M	1	0	0	1	0	0	0	1	0	0	1	0	0	1	0	0	2	0	0	2	0	0	3	0	0	1	0	0	1	0	0	1	0	0	2	0	0
	4		M	1	0	0	1	0	0	0	1	0	0	1	0	0	1	0	0	2	0	0	2	0	0	3	0	0	1	0	0	1	0	0	1	0	0	2	0	0
T45	1	FLUORESCENT	F	1	0	0	1	0	0	0	1	0	0	1	0	0	1	0	0	2	0	0	2	0	0	3	0	0	1	0	0	1	0	0	1	0	0	2	0	0
	2		F	1	0	0	1	0	0	0	1	0	0	1	0	0	1	0	0	2	0	0	2	0	0	3	0	0	1	0	0	1	0	0	1	0	0	2	0	0
	3		F	1	0	0	1	0	0	0	1	0	0	1	0	0	1	0	0	2	0	0	2	0	0	3	0	0	1	0	0	1	0	0	1	0	0	2	0	0
	4		M	1	0	0	1	0	0	0	1	0	0	1	0	0	1	0	0	2	0	0	2	0	0	3	0	0	1	0	0	1	0	0	1	0	0	2	0	0
	5		M	1	0	0	1	0	0	0	1	0	0	1	0	0	1	0	0	2	0	0	2	0	0	3	0	0	1	0	0	1	0	0	1	0	0	2	0	0
	6		M	1	0	0	1	0	0	0	1	0	0	1	0	0	1	0	0	2	0	0	2	0	0	3	0	0	1	0	0	1	0	0	1	0	0	2	0	0
	1	COSMETIC	F	1	0	0	1	0	0	0	1	0	0	1	0	0	1	0	0	2	0	0	2	0	0	3	0	0	1	0	0	1	0	0	1	0	0	2	0	0
	2		F	1	0	0	1	0	0	0	1	0	0	1	0	0	1	0	0	2	0	0	2	0	0	3	0	0	1	0	0	1	0	0	1	0	0	2	0	0
	3		F	1	0	0	1	0	0	0	1	0	0	1	0	0	1	0	0	2	0	0	2	0	0	3	0	0	1	0	0	1	0	0	1	0	0	2	0	0
	4		M	1	0	0	1	0	0	0	1	0	0	1	0	0	1	0	0	2	0	0	2	0	0	3	0	0	1	0	0	1	0	0	1	0	0	2	0	0
	5		M	1	0	0	1	0	0	0	1	0	0	1	0	0	1	0	0	2	0	0	2	0	0	3	0	0	1	0	0	1	0	0	1	0	0	2	0	0
	6		M	1	0	0	1	0	0	0	1	0	0	1	0	0	1	0	0	2	0	0	2	0	0	3	0	0	1	0	0	1	0	0	1	0	0	2	0	0
	1	CONTROL	F	1	0	0	1	0	0	0	1	0	0	1	0	0	1	0	0	2	0	0	2	0	0	3	0	0	1	0	0	1	0	0	1	0	0	2	0	0
	2		F	1	0	0	1	0	0	0	1	0	0	1	0	0	1	0	0	2	0	0	2	0	0	3	0	0	1	0	0	1	0	0	1	0	0	2	0	0
	3		M	1	0	0	1	0	0	0	1	0	0	1	0	0	1	0	0	2	0	0	2	0	0	3	0	0	1	0	0	1	0	0	1	0	0	2	0	0
	4		M	1	0	0	1	0	0	0	1	0	0	1	0	0	1	0	0	2	0	0	2	0	0	3	0	0	1	0	0	1	0	0	1	0	0	2	0	0
DEPURATION	1	FLUORESCENT	F	1	0	0	1	0	0	0	1	0	0	1	0	0	1	0	0	2	0	0	2	0	0	3	0	0	1	0	0	1	0	0	1	0	0	2	0	0
	2		F	1	0	0	1	0	0	0	1	0	0	1	0	0	1	0	0	2	0	0	2	0	0	3	0	0	1	0	0	1	0	0	1	0	0	2	0	0
	3		F	1	0	0	1	0	0	0	1	0	0	1	0	0	1	0	0	2	0	0	2	0	0	3	0	0	1	0	0	1	0	0	1	0	0	2	0	0
	4		M	1	0	0	1	0	0	0	1	0	0	1	0	0	1	0	0	2	0	0	2	0	0	3	0	0	1	0	0	1	0	0	1	0	0	2	0	0
	5		M	1	0	0	1	0	0	0	1	0	0	1	0	0	1	0	0	2	0	0	2	0	0	3	0	0	1	0	0	1	0	0	1	0	0	2	0	0
	6		M	1	0	0	1	0	0	0	1	0	0	1	0	0	1	0	0	2	0	0	2	0	0	3	0	0	1	0	0	1	0	0	1	0	0	2	0	0
	1	COSMETIC	F	1	0	0	1	0	0	0	1	0	0	1	0	0	1	0	0	2	0	0	2	0	0	3	0	0	1	0	0	1	0	0	1	0	0	2	0	0
	2		F	1	0	0	1	0	0	0	1	0	0	1	0	0	1	0	0	2	0	0	2	0	0	3	0	0	1	0	0	1	0	0	1	0	0	2	0	0
	3		F	1	0	0	1	0	0	0	1	0	0	1	0	0	1	0	0	2	0	0	2	0	0	3	0	0	1	0	0	1	0	0	1	0	0	2	0	0
	4		M	1	0	0	1	0	0	0	1	0	0	1	0	0	1	0	0	2	0	0	2	0	0	3	0	0	1	0	0	1	0	0	1	0	0	2	0	0
	5		M	1	0	0	1	0	0	0	1	0	0	1	0	0	1	0	0	2	0	0	2	0	0	3	0	0	1	0	0	1	0	0	1	0	0	2	0	0
	6		M	1	0	0	1	0	0	0	1	0	0	1	0	0	1	0	0	2	0	0	2	0	0	3	0	0	1	0	0	1	0	0	1	0	0	2	0	0

**T0**	Beginning of the experiment.
**T30**	30 days after the beginning of the experiment.
**T45**	45 days after the beginning of the experiment.
**F**	Female
**M**	Male
**a**	Score value
**w**	Importance factor
**rp**	Reaction pattern
**I org**	Organ index (is the sum of the multiplied importance factors and score values of all changes found within the examined organ).
**I org rp**	Reaction index of an organ (is the sum of the multiplied importance factors and score values of the alterations of the corresponding reaction patterns).

							Progressive Changes (rp3)													Inflammation (rp4)										Tumor (rp5)							I org
							Epithelium						*Lamina Propria*																								
Atrophy			Necrosis			I org rp	Hypertrophy			Hyperplasia			Hypertrophy			Hyperplasia			I org rp	Exudate			Activation of RES			Infiltration			I org rp	Benign			Malignant			I org rp	
w	a	a x w	w	a	a x w		w	a	a x w	w	a	a x w	w	a	a x w	w	a	a x w		w	a	a x w	w	a	a x w	w	a	a x w		w	a	a x w	w	a	a x w		
2	0	0	3	0	0	0	1	0	0	2	0	0	1	0	0	2	0	0	0	1	0	0	1	0	0	2	0	0	0	2	0	0	3	0	0	0	0
2	0	0	3	0	0	0	1	0	0	2	0	0	1	0	0	2	0	0	0	1	0	0	1	0	0	2	0	0	0	2	0	0	3	0	0	0	0
2	0	0	3	0	0	0	1	0	0	2	0	0	1	0	0	2	0	0	0	1	0	0	1	0	0	2	0	0	0	2	0	0	3	0	0	0	0
2	0	0	3	0	0	0	1	0	0	2	0	0	1	0	0	2	0	0	0	1	0	0	1	0	0	2	0	0	0	2	0	0	3	0	0	0	0
2	0	0	3	0	0	0	1	0	0	2	0	0	1	0	0	2	0	0	0	1	0	0	1	0	0	2	0	0	0	2	0	0	3	0	0	0	0
2	0	0	3	0	0	0	1	0	0	2	0	0	1	0	0	2	0	0	0	1	0	0	1	0	0	2	0	0	0	2	0	0	3	0	0	0	0
2	0	0	3	0	0	0	1	0	0	2	0	0	1	0	0	2	0	0	0	1	0	0	1	0	0	2	0	0	0	2	0	0	3	0	0	0	0
2	0	0	3	0	0	0	1	0	0	2	0	0	1	0	0	2	0	0	0	1	0	0	1	0	0	2	0	0	0	2	0	0	3	0	0	0	0
2	0	0	3	0	0	0	1	0	0	2	0	0	1	0	0	2	0	0	0	1	0	0	1	0	0	2	0	0	0	2	0	0	3	0	0	0	0
2	0	0	3	0	0	0	1	0	0	2	0	0	1	0	0	2	0	0	0	1	0	0	1	0	0	2	0	0	0	2	0	0	3	0	0	0	0
2	0	0	3	0	0	0	1	0	0	2	0	0	1	0	0	2	0	0	0	1	0	0	1	0	0	2	0	0	0	2	0	0	3	0	0	0	0
2	0	0	3	0	0	0	1	0	0	2	0	0	1	0	0	2	0	0	0	1	0	0	1	0	0	2	0	0	0	2	0	0	3	0	0	0	0
2	0	0	3	0	0	0	1	0	0	2	0	0	1	0	0	2	0	0	0	1	0	0	1	0	0	2	0	0	0	2	0	0	3	0	0	0	0
2	0	0	3	0	0	0	1	0	0	2	0	0	1	0	0	2	0	0	0	1	0	0	1	0	0	2	0	0	0	2	0	0	3	0	0	0	0
2	0	0	3	0	0	0	1	0	0	2	0	0	1	0	0	2	0	0	0	1	0	0	1	0	0	2	0	0	0	2	0	0	3	0	0	0	0
2	0	0	3	0	0	0	1	0	0	2	0	0	1	0	0	2	0	0	0	1	0	0	1	0	0	2	0	0	0	2	0	0	3	0	0	0	0
2	0	0	3	0	0	0	1	0	0	2	0	0	1	0	0	2	0	0	0	1	0	0	1	0	0	2	0	0	0	2	0	0	3	0	0	0	0
2	0	0	3	0	0	0	1	0	0	2	0	0	1	0	0	2	0	0	0	1	0	0	1	0	0	2	0	0	0	2	0	0	3	0	0	0	0
2	0	0	3	0	0	0	1	0	0	2	0	0	1	0	0	2	0	0	0	1	0	0	1	0	0	2	0	0	0	2	0	0	3	0	0	0	0
2	0	0	3	0	0	0	1	0	0	2	0	0	1	0	0	2	0	0	0	1	0	0	1	0	0	2	0	0	0	2	0	0	3	0	0	0	0
2	0	0	3	0	0	0	1	0	0	2	0	0	1	0	0	2	0	0	0	1	0	0	1	0	0	2	0	0	0	2	0	0	3	0	0	0	0
2	0	0	3	0	0	0	1	0	0	2	0	0	1	0	0	2	0	0	0	1	0	0	1	0	0	2	0	0	0	2	0	0	3	0	0	0	0
2	0	0	3	0	0	0	1	0	0	2	0	0	1	0	0	2	0	0	0	1	0	0	1	0	0	2	0	0	0	2	0	0	3	0	0	0	0
2	0	0	3	0	0	0	1	0	0	2	0	0	1	0	0	2	0	0	0	1	0	0	1	0	0	2	0	0	0	2	0	0	3	0	0	0	0
2	0	0	3	0	0	0	1	0	0	2	0	0	1	0	0	2	0	0	0	1	0	0	1	0	0	2	0	0	0	2	0	0	3	0	0	0	0
2	0	0	3	0	0	0	1	0	0	2	0	0	1	0	0	2	0	0	0	1	0	0	1	0	0	2	0	0	0	2	0	0	3	0	0	0	0
2	0	0	3	0	0	0	1	0	0	2	0	0	1	0	0	2	0	0	0	1	0	0	1	0	0	2	0	0	0	2	0	0	3	0	0	0	0
2	0	0	3	0	0	0	1	0	0	2	0	0	1	0	0	2	0	0	0	1	0	0	1	0	0	2	0	0	0	2	0	0	3	0	0	0	0
2	0	0	3	0	0	0	1	0	0	2	0	0	1	0	0	2	0	0	0	1	0	0	1	0	0	2	0	0	0	2	0	0	3	0	0	0	0
2	0	0	3	0	0	0	1	0	0	2	0	0	1	0	0	2	0	0	0	1	0	0	1	0	0	2	0	0	0	2	0	0	3	0	0	0	0
2	0	0	3	0	0	0	1	0	0	2	0	0	1	0	0	2	0	0	0	1	0	0	1	0	0	2	0	0	0	2	0	0	3	0	0	0	0
2	0	0	3	0	0	0	1	0	0	2	0	0	1	0	0	2	0	0	0	1	0	0	1	0	0	2	0	0	0	2	0	0	3	0	0	0	0
2	0	0	3	0	0	0	1	0	0	2	0	0	1	0	0	2	0	0	0	1	0	0	1	0	0	2	0	0	0	2	0	0	3	0	0	0	0
2	0	0	3	0	0	0	1	0	0	2	0	0	1	0	0	2	0	0	0	1	0	0	1	0	0	2	0	0	0	2	0	0	3	0	0	0	0
2	0	0	3	0	0	0	1	0	0	2	0	0	1	0	0	2	0	0	0	1	0	0	1	0	0	2	0	0	0	2	0	0	3	0	0	0	0
2	0	0	3	0	0	0	1	0	0	2	0	0	1	0	0	2	0	0	0	1	0	0	1	0	0	2	0	0	0	2	0	0	3	0	0	0	0
2	0	0	3	0	0	0	1	0	0	2	0	0	1	0	0	2	0	0	0	1	0	0	1	0	0	2	0	0	0	2	0	0	3	0	0	0	0
2	0	0	3	0	0	0	1	0	0	2	0	0	1	0	0	2	0	0	0	1	0	0	1	0	0	2	0	0	0	2	0	0	3	0	0	0	0
2	0	0	3	0	0	0	1	0	0	2	0	0	1	0	0	2	0	0	0	1	0	0	1	0	0	2	0	0	0	2	0	0	3	0	0	0	0
2	0	0	3	0	0	0	1	0	0	2	0	0	1	0	0	2	0	0	0	1	0	0	1	0	0	2	0	0	0	2	0	0	3	0	0	0	0
2	0	0	3	0	0	0	1	0	0	2	0	0	1	0	0	2	0	0	0	1	0	0	1	0	0	2	0	0	0	2	0	0	3	0	0	0	0
2	0	0	3	0	0	0	1	0	0	2	0	0	1	0	0	2	0	0	0	1	0	0	1	0	0	2	0	0	0	2	0	0	3	0	0	0	0
2	0	0	3	0	0	0	1	0	0	2	0	0	1	0	0	2	0	0	0	1	0	0	1	0	0	2	0	0	0	2	0	0	3	0	0	0	0
2	0	0	3	0	0	0	1	0	0	2	0	0	1	0	0	2	0	0	0	1	0	0	1	0	0	2	0	0	0	2	0	0	3	0	0	0	0
2	0	0	3	0	0	0	1	0	0	2	0	0	1	0	0	2	0	0	0	1	0	0	1	0	0	2	0	0	0	2	0	0	3	0	0	0	0
2	0	0	3	0	0	0	1	0	0	2	0	0	1	0	0	2	0	0	0	1	0	0	1	0	0	2	0	0	0	2	0	0	3	0	0	0	0
2	0	0	3	0	0	0	1	0	0	2	0	0	1	0	0	2	0	0	0	1	0	0	1	0	0	2	0	0	0	2	0	0	3	0	0	0	0
2	0	0	3	0	0	0	1	0	0	2	0	0	1	0	0	2	0	0	0	1	0	0	1	0	0	2	0	0	0	2	0	0	3	0	0	0	0
2	0	0	3	0	0	0	1	0	0	2	0	0	1	0	0	2	0	0	0	1	0	0	1	0	0	2	0	0	0	2	0	0	3	0	0	0	0

Supplementary Table S2. Histopathological assessment tools for liver

				Circulatory Disturbances (rp1)							Regressive changes (rp2)																																							
											Liver tissue																						*Interstitial tissue*																	
	D. rerio	Test Group	Sex	Haemorrhage / hyperaemia / aneurysm			Intercelular oedema			I org rp	Architectural and strucutral alterations			Plasma alterations			Deposits			Decreased hepatocellular vacuolation			Nuclear alterations			Atrophy			Necrosis			Degree of vacuolation	Architectural and strucutral alterations			Plasma alterations			Deposits			Nuclear alterations			Atrophy			Necrosis		
				w	a	a x w	w	a	a x w		w	a	a x w	w	a	a x w	w	a	a x w	w	a	a x w	w	a	a x w	w	a	a x w	w	a	a x w		w	a	a x w	w	a	a x w	w	a	a x w	w	a	a x w	w	a	a x w	w	a	a x w
T0	1	CONTROL	F	1	0	0	1	0	0	0	1	0	0	1	0	0	1	0	0	1	0	0	2	0	0	2	0	0	3	0	0	3	1	0	0	1	0	0	1	0	0	2	0	0	2	0	0	3	0	0
	2		F	1	0	0	1	0	0	0	1	0	0	1	0	0	1	0	0	1	0	0	2	0	0	2	0	0	3	0	0	3	1	0	0	1	0	0	1	0	0	2	0	0	2	0	0	3	0	0
	3		M	1	0	0	1	0	0	0	1	0	0	1	0	0	1	0	0	1	0	0	2	0	0	2	0	0	3	0	0	3	1	0	0	1	0	0	1	0	0	2	0	0	2	0	0	3	0	0
	4		M	1	0	0	1	0	0	0	1	0	0	1	0	0	1	0	0	1	0	0	2	0	0	2	0	0	3	0	0	3	1	0	0	1	0	0	1	0	0	2	0	0	2	0	0	3	0	0
T30	1	FLUORESCENT	F	1	0	0	1	0	0	0	1	0	0	1	0	0	1	0	0	1	2	2	2	0	0	2	0	0	3	0	0	1	1	0	0	1	0	0	1	0	0	2	0	0	2	0	0	3	0	0
	2		F	1	0	0	1	0	0	0	1	0	0	1	2	2	1	0	0	1	0	0	2	0	0	2	0	0	3	0	0	4	1	0	0	1	0	0	1	0	0	2	0	0	2	0	0	3	0	0
	3		F	1	0	0	1	0	0	0	1	0	0	1	0	0	1	0	0	1	0	0	2	0	0	2	0	0	3	0	0	3	1	0	0	1	0	0	1	0	0	2	0	0	2	0	0	3	0	0
	4		M	1	0	0	1	0	0	0	1	0	0	1	0	0	1	0	0	1	0	0	2	0	0	2	0	0	3	0	0	3	1	0	0	1	0	0	1	0	0	2	0	0	2	0	0	3	0	0
	5		M	1	0	0	1	0	0	0	1	0	0	1	0	0	1	0	0	1	0	0	2	0	0	2	0	0	3	0	0	3	1	0	0	1	0	0	1	0	0	2	0	0	2	0	0	3	0	0
	6		M	1	0	0	1	0	0	0	1	0	0	1	0	0	1	0	0	1	0	0	2	0	0	2	0	0	3	0	0	3	1	0	0	1	0	0	1	0	0	2	0	0	2	0	0	3	0	0
	1	COSMETIC	F	1	0	0	1	0	0	0	1	0	0	1	2	2	1	0	0	1	0	0	2	0	0	2	0	0	3	0	0	4	1	0	0	1	0	0	1	0	0	2	0	0	2	0	0	3	0	0
	2		F	1	0	0	1	0	0	0	1	0	0	1	2	2	1	0	0	1	0	0	2	0	0	2	0	0	3	0	0	4	1	0	0	1	0	0	1	0	0	2	0	0	2	0	0	3	0	0
	3		F	1	0	0	1	0	0	0	1	0	0	1	2	2	1	0	0	1	0	0	2	0	0	2	0	0	3	0	0	4	1	0	0	1	0	0	1	0	0	2	0	0	2	0	0	3	0	0
	4		M	1	0	0	1	0	0	0	1	0	0	1	0	0	1	0	0	1	0	0	2	0	0	2	0	0	3	0	0	3	1	0	0	1	0	0	1	0	0	2	0	0	2	0	0	3	0	0
	5		M	1	0	0	1	0	0	0	1	0	0	1	0	0	1	0	0	1	0	0	2	0	0	2	0	0	3	0	0	3	1	0	0	1	0	0	1	0	0	2	0	0	2	0	0	3	0	0
	6		M	1	0	0	1	0	0	0	1	0	0	1	0	0	1	0	0	1	0	0	2	0	0	2	0	0	3	0	0	3	1	0	0	1	0	0	1	0	0	2	0	0	2	0	0	3	0	0
	1	CONTROL	F	1	0	0	1	0	0	0	1	0	0	1	0	0	1	0	0	1	0	0	2	0	0	2	0	0	3	0	0	3	1	0	0	1	0	0	1	0	0	2	0	0	2	0	0	3	0	0
	2		F	1	0	0	1	0	0	0	1	0	0	1	0	0	1	0	0	1	0	0	2	0	0	2	0	0	3	0	0	3	1	0	0	1	0	0	1	0	0	2	0	0	2	0	0	3	0	0
	3		M	1	0	0	1	0	0	0	1	0	0	1	0	0	1	0	0	1	0	0	2	0	0	2	0	0	3	0	0	3	1	0	0	1	0	0	1	0	0	2	0	0	2	0	0	3	0	0
	4		M	1	0	0	1	0	0	0	1	0	0	1	0	0	1	0	0	1	0	0	2	0	0	2	0	0	3	0	0	3	1	0	0	1	0	0	1	0	0	2	0	0	2	0	0	3	0	0
T45	1	FLUORESCENT	F	1	0	0	1	0	0	0	1	0	0	1	0	0	1	0	0	1	0	0	2	0	0	2	0	0	3	0	0	3	1	0	0	1	0	0	1	0	0	2	0	0	2	0	0	3	0	0
	2		F	1	0	0	1	0	0	0	1	0	0	1	0	0	1	0	0	1	1	1	2	0	0	2	0	0	3	0	0	2	1	0	0	1	0	0	1	0	0	2	0	0	2	0	0	3	0	0
	3		F	1	0	0	1	0	0	0	1	0	0	1	0	0	1	0	0	1	0	0	2	0	0	2	0	0	3	0	0	3	1	0	0	1	0	0	1	0	0	2	0	0	2	0	0	3	0	0
	4		M	1	0	0	1	0	0	0	1	0	0	1	0	0	1	0	0	1	0	0	2	0	0	2	0	0	3	0	0	3	1	0	0	1	0	0	1	0	0	2	0	0	2	0	0	3	0	0
	5		M	1	0	0	1	0	0	0	1	0	0	1	0	0	1	0	0	1	0	0	2	0	0	2	0	0	3	0	0	3	1	0	0	1	0	0	1	0	0	2	0	0	2	0	0	3	0	0
	6		M	1	0	0	1	0	0	0	1	0	0	1	0	0	1	0	0	1	0	0	2	0	0	2	0	0	3	0	0	3	1	0	0	1	0	0	1	0	0	2	0	0	2	0	0	3	0	0
	1	COSMETIC	F	1	0	0	1	0	0	0	1	0	0	1	0	0	1	0	0	1	0	0	2	0	0	2	0	0	3	0	0	3	1	0	0	1	0	0	1	0	0	2	0	0	2	0	0	3	0	0
	2		F	1	0	0	1	0	0	0	1	0	0	1	0	0	1	0	0	1	0	0	2	0	0	2	0	0	3	0	0	3	1	0	0	1	0	0	1	0	0	2	0	0	2	0	0	3	0	0
	3		F	1	0	0	1	0	0	0	1	0	0	1	2	2	1	0	0	1	0	0	2	0	0	2	0	0	3	0	0	4	1	0	0	1	0	0	1	0	0	2	0	0	2	0	0	3	0	0
	4		M	1	0	0	1	0	0	0	1	0	0	1	0	0	1	0	0	1	0	0	2	0	0	2	0	0	3	0	0	3	1	0	0	1	0	0	1	0	0	2	0	0	2	0	0	3	0	0
	5		M	1	0	0	1	0	0	0	1	0	0	1	0	0	1	0	0	1	0	0	2	0	0	2	0	0	3	0	0	3	1	0	0	1	0	0	1	0	0	2	0	0	2	0	0	3	0	0
	6		M	1	0	0	1	0	0	0	1	0	0	1	0	0	1	0	0	1	0	0	2	0	0	2	0	0	3	0	0	3	1	0	0	1	0	0	1	0	0	2	0	0	2	0	0	3	0	0
	1	CONTROL	F	1	0	0	1	0	0	0	1	0	0	1	0	0	1	0	0	1	0	0	2	0	0	2	0	0	3	0	0	3	1	0	0	1	0	0	1	0	0	2	0	0	2	0	0	3	0	0
	2		F	1	0	0	1	0	0	0	1	0	0	1	0	0	1	0	0	1	0	0	2	0	0	2	0	0	3	0	0	3	1	0	0	1	0	0	1	0	0	2	0	0	2	0	0	3	0	0
	3		M	1	0	0	1	0	0	0	1	0	0	1	0	0	1	0	0	1	0	0	2	0	0	2	0	0	3	0	0	3	1	0	0	1	0	0	1	0	0	2	0	0	2	0	0	3	0	0
	4		M	1	0	0	1	0	0	0	1	0	0	1	0	0	1	0	0	1	0	0	2	0	0	2	0	0	3	0	0	3	1	0	0	1	0	0	1	0	0	2	0	0	2	0	0	3	0	0
DEPURATION	1	FLUORESCENT	F	1	0	0	1	0	0	0	1	0	0	1	0	0	1	0	0	1	1	1	2	0	0	2	0	0	3	0	0	2	1	0	0	1	0	0	1	0	0	2	0	0	2	0	0	3	0	0
	2		F	1	0	0	1	0	0	0	1	0	0	1	0	0	1	0	0	1	0	0	2	0	0	2	0	0	3	0	0	3	1	0	0	1	0	0	1	0	0	2	0	0	2	0	0	3	0	0
	3		F	1	0	0	1	0	0	0	1	0	0	1	2	2	1	0	0	1	0	0	2	0	0	2	0	0	3	0	0	4	1	0	0	1	0	0	1	0	0	2	0	0	2	0	0	3	0	0
	4		M	1	0	0	1	0	0	0	1	0	0	1	0	0	1	0	0	1	1	1	2	0	0	2	0	0	3	0	0	2	1	0	0	1	0	0	1	0	0	2	0	0	2	0	0	3	0	0
	5		M	1	0	0	1	0	0	0	1	0	0	1	0	0	1	0	0	1	1	1	2	0	0	2	0	0	3	0	0	2	1	0	0	1	0	0	1	0	0	2	0	0	2	0	0	3	0	0
	6		M	1	0	0	1	0	0	0	1	0	0	1	0	0	1	0	0	1	0	0	2	0	0	2	0	0	3	0	0	3	1	0	0	1	0	0	1	0	0	2	0	0	2	0	0	3	0	0
	1	COSMETIC	F	1	0	0	1	0	0	0	1	0	0	1	0	0	1	0	0	1	0	0	2	0	0	2	0	0	3	0	0	3	1	0	0	1	0	0	1	0	0	2	0	0	2	0	0	3	0	0
	2		F	1	0	0	1	0	0	0	1	0	0	1	0	0	1	0	0	1	1	1	2	0	0	2	0	0	3	0	0	2	1	0	0	1	0	0	1	0	0	2	0	0	2	0	0	3	0	0
	3		F	1	0	0	1	0	0	0	1	0	0	1	0	0	1	0	0	1	0	0	2	0	0	2	0	0	3	0	0	3	1	0	0	1	0	0	1	0	0	2	0	0	2	0	0	3	0	0
	4		M	1	0	0	1	0	0	0	1	0	0	1	0	0	1	0	0	1	2	2	2	0	0	2	0	0	3	0	0	1	1	0	0	1	0	0	1	0	0	2	0	0	2	0	0	3	0	0
	5		M	1	0	0	1	0	0	0	1	0	0	1	0	0	1	0	0	1	2	2	2	0	0	2	0	0	3	0	0	1	1	0	0	1	0	0	1	0	0	2	0	0	2	0	0	3	0	0
	6		M	1	0	0	1	0	0	0	1	0	0	1	0	0	1	0	0	1	1	1	2	0	0	2	0	0	3	0	0	2	1	0	0	1	0	0	1	0	0	2	0	0	2	0	0	3	0	0

**T0**	Beginning of the experiment.
**T30**	30 days after the beginning of the experiment.
**T45**	45 days after the beginning of the experiment.
**F**	Female
**M**	Male
**a**	Score value
**w**	Importance factor
**rp**	Reaction pattern
**I org**	Organ index (is the sum of the multiplied importance factors and score values of all changes found within the examined organ).
**I org rp**	Reaction index of an organ (is the sum of the multiplied importance factors and score values of the alterations of the corresponding reaction patterns).

																		Progressive Changes (rp3)																				Inflammation (rp4)										Tumor (rp5)							I org
Bile Duct																		I org rp	*Liver tissue*						*Interstitial tissue*						*Bile Duct*						I org rp																		
Architectural and strucutral alterations			Plasma alterations			Deposits			Nuclear alterations			Atrophy			Necrosis				Hypertrophy			Hyperplasia			Hypertrophy			Hyperplasia			Hypertrophy			Hyperplasia				Exudate			Activation of RES			Infiltration			I org rp	Benign			Malignant			I org rp	
w	a	a x w	w	a	a x w	w	a	a x w	w	a	a x w	w	a	a x w	w	a	a x w		w	a	a x w	w	a	a x w	w	a	a x w	w	a	a x w	w	a	a x w	w	a	a x w		w	a	a x w	w	a	a x w	w	a	a x w		w	a	a x w	w	a	a x w		
1	0	0	1	0	0	1	0	0	2	0	0	2	0	0	3	0	0	0	1	0	0	2	0	0	1	0	0	2	0	0	1	0	0	2	0	0	0	1	0	0	1	0	0	2	0	0	0	2	0	0	3	0	0	0	0
1	0	0	1	0	0	1	0	0	2	0	0	2	0	0	3	0	0	0	1	0	0	2	0	0	1	0	0	2	0	0	1	0	0	2	0	0	0	1	0	0	1	0	0	2	0	0	0	2	0	0	3	0	0	0	0
1	0	0	1	0	0	1	0	0	2	0	0	2	0	0	3	0	0	0	1	0	0	2	0	0	1	0	0	2	0	0	1	0	0	2	0	0	0	1	0	0	1	0	0	2	0	0	0	2	0	0	3	0	0	0	0
1	0	0	1	0	0	1	0	0	2	0	0	2	0	0	3	0	0	0	1	0	0	2	0	0	1	0	0	2	0	0	1	0	0	2	0	0	0	1	0	0	1	0	0	2	0	0	0	2	0	0	3	0	0	0	0
1	0	0	1	0	0	1	0	0	2	0	0	2	0	0	3	0	0	0	1	0	0	2	0	0	1	0	0	2	0	0	1	0	0	2	0	0	0	1	0	0	1	0	0	2	0	0	0	2	0	0	3	0	0	0	2
1	0	0	1	0	0	1	0	0	2	0	0	2	0	0	3	0	0	2	1	0	0	2	0	0	1	0	0	2	0	0	1	0	0	2	0	0	0	1	0	0	1	0	0	2	0	0	0	2	0	0	3	0	0	0	2
1	0	0	1	0	0	1	0	0	2	0	0	2	0	0	3	0	0	0	1	0	0	2	0	0	1	0	0	2	0	0	1	0	0	2	0	0	0	1	0	0	1	0	0	2	0	0	0	2	0	0	3	0	0	0	0
1	0	0	1	0	0	1	0	0	2	0	0	2	0	0	3	0	0	0	1	0	0	2	0	0	1	0	0	2	0	0	1	0	0	2	0	0	0	1	0	0	1	0	0	2	0	0	0	2	0	0	3	0	0	0	0
1	0	0	1	0	0	1	0	0	2	0	0	2	0	0	3	0	0	0	1	0	0	2	0	0	1	0	0	2	0	0	1	0	0	2	0	0	0	1	0	0	1	0	0	2	0	0	0	2	0	0	3	0	0	0	0
1	0	0	1	0	0	1	0	0	2	0	0	2	0	0	3	0	0	0	1	0	0	2	0	0	1	0	0	2	0	0	1	0	0	2	0	0	0	1	0	0	1	0	0	2	0	0	0	2	0	0	3	0	0	0	0
1	0	0	1	0	0	1	0	0	2	0	0	2	0	0	3	0	0	2	1	0	0	2	0	0	1	0	0	2	0	0	1	0	0	2	0	0	0	1	0	0	1	0	0	2	0	0	0	2	0	0	3	0	0	0	2
1	0	0	1	0	0	1	0	0	2	0	0	2	0	0	3	0	0	2	1	0	0	2	0	0	1	0	0	2	0	0	1	0	0	2	0	0	0	1	0	0	1	0	0	2	0	0	0	2	0	0	3	0	0	0	2
1	0	0	1	0	0	1	0	0	2	0	0	2	0	0	3	0	0	2	1	0	0	2	0	0	1	0	0	2	0	0	1	0	0	2	0	0	0	1	0	0	1	0	0	2	0	0	0	2	0	0	3	0	0	0	2
1	0	0	1	0	0	1	0	0	2	0	0	2	0	0	3	0	0	0	1	0	0	2	0	0	1	0	0	2	0	0	1	0	0	2	0	0	0	1	0	0	1	0	0	2	0	0	0	2	0	0	3	0	0	0	0
1	0	0	1	0	0	1	0	0	2	0	0	2	0	0	3	0	0	0	1	0	0	2	0	0	1	0	0	2	0	0	1	0	0	2	0	0	0	1	0	0	1	0	0	2	0	0	0	2	0	0	3	0	0	0	0
1	0	0	1	0	0	1	0	0	2	0	0	2	0	0	3	0	0	0	1	0	0	2	0	0	1	0	0	2	0	0	1	0	0	2	0	0	0	1	0	0	1	0	0	2	0	0	0	2	0	0	3	0	0	0	0
1	0	0	1	0	0	1	0	0	2	0	0	2	0	0	3	0	0	0	1	0	0	2	0	0	1	0	0	2	0	0	1	0	0	2	0	0	0	1	0	0	1	0	0	2	0	0	0	2	0	0	3	0	0	0	0
1	0	0	1	0	0	1	0	0	2	0	0	2	0	0	3	0	0	0	1	0	0	2	0	0	1	0	0	2	0	0	1	0	0	2	0	0	0	1	0	0	1	0	0	2	0	0	0	2	0	0	3	0	0	0	0
1	0	0	1	0	0	1	0	0	2	0	0	2	0	0	3	0	0	0	1	0	0	2	0	0	1	0	0	2	0	0	1	0	0	2	0	0	0	1	0	0	1	0	0	2	0	0	0	2	0	0	3	0	0	0	0
1	0	0	1	0	0	1	0	0	2	0	0	2	0	0	3	0	0	0	1	0	0	2	0	0	1	0	0	2	0	0	1	0	0	2	0	0	0	1	0	0	1	0	0	2	0	0	0	2	0	0	3	0	0	0	0
1	0	0	1	0	0	1	0	0	2	0	0	2	0	0	3	0	0	0	1	0	0	2	0	0	1	0	0	2	0	0	1	0	0	2	0	0	0	1	0	0	1	0	0	2	0	0	0	2	0	0	3	0	0	0	1
1	0	0	1	0	0	1	0	0	2	0	0	2	0	0	3	0	0	0	1	0	0	2	0	0	1	0	0	2	0	0	1	0	0	2	0	0	0	1	0	0	1	0	0	2	0	0	0	2	0	0	3	0	0	0	0
1	0	0	1	0	0	1	0	0	2	0	0	2	0	0	3	0	0	0	1	0	0	2	0	0	1	0	0	2	0	0	1	0	0	2	0	0	0	1	0	0	1	0	0	2	0	0	0	2	0	0	3	0	0	0	0
1	0	0	1	0	0	1	0	0	2	0	0	2	0	0	3	0	0	0	1	0	0	2	0	0	1	0	0	2	0	0	1	0	0	2	0	0	0	1	0	0	1	0	0	2	0	0	0	2	0	0	3	0	0	0	0
1	0	0	1	0	0	1	0	0	2	0	0	2	0	0	3	0	0	0	1	0	0	2	0	0	1	0	0	2	0	0	1	0	0	2	0	0	0	1	0	0	1	0	0	2	0	0	0	2	0	0	3	0	0	0	0
1	0	0	1	0	0	1	0	0	2	0	0	2	0	0	3	0	0	0	1	0	0	2	0	0	1	0	0	2	0	0	1	0	0	2	0	0	0	1	0	0	1	0	0	2	0	0	0	2	0	0	3	0	0	0	0
1	0	0	1	0	0	1	0	0	2	0	0	2	0	0	3	0	0	0	1	0	0	2	0	0	1	0	0	2	0	0	1	0	0	2	0	0	0	1	0	0	1	0	0	2	0	0	0	2	0	0	3	0	0	0	0
1	0	0	1	0	0	1	0	0	2	0	0	2	0	0	3	0	0	2	1	0	0	2	0	0	1	0	0	2	0	0	1	0	0	2	0	0	0	1	0	0	1	0	0	2	0	0	0	2	0	0	3	0	0	0	2
1	0	0	1	0	0	1	0	0	2	0	0	2	0	0	3	0	0	0	1	0	0	2	0	0	1	0	0	2	0	0	1	0	0	2	0	0	0	1	0	0	1	0	0	2	0	0	0	2	0	0	3	0	0	0	0
1	0	0	1	0	0	1	0	0	2	0	0	2	0	0	3	0	0	0	1	0	0	2	0	0	1	0	0	2	0	0	1	0	0	2	0	0	0	1	0	0	1	0	0	2	0	0	0	2	0	0	3	0	0	0	0
1	0	0	1	0	0	1	0	0	2	0	0	2	0	0	3	0	0	0	1	0	0	2	0	0	1	0	0	2	0	0	1	0	0	2	0	0	0	1	0	0	1	0	0	2	0	0	0	2	0	0	3	0	0	0	0
1	0	0	1	0	0	1	0	0	2	0	0	2	0	0	3	0	0	0	1	0	0	2	0	0	1	0	0	2	0	0	1	0	0	2	0	0	0	1	0	0	1	0	0	2	0	0	0	2	0	0	3	0	0	0	0
1	0	0	1	0	0	1	0	0	2	0	0	2	0	0	3	0	0	0	1	0	0	2	0	0	1	0	0	2	0	0	1	0	0	2	0	0	0	1	0	0	1	0	0	2	0	0	0	2	0	0	3	0	0	0	0
1	0	0	1	0	0	1	0	0	2	0	0	2	0	0	3	0	0	0	1	0	0	2	0	0	1	0	0	2	0	0	1	0	0	2	0	0	0	1	0	0	1	0	0	2	0	0	0	2	0	0	3	0	0	0	0
1	0	0	1	0	0	1	0	0	2	0	0	2	0	0	3	0	0	0	1	0	0	2	0	0	1	0	0	2	0	0	1	0	0	2	0	0	0	1	0	0	1	0	0	2	0	0	0	2	0	0	3	0	0	0	1
1	0	0	1	0	0	1	0	0	2	0	0	2	0	0	3	0	0	0	1	0	0	2	0	0	1	0	0	2	0	0	1	0	0	2	0	0	0	1	0	0	1	0	0	2	0	0	0	2	0	0	3	0	0	0	0
1	0	0	1	0	0	1	0	0	2	0	0	2	0	0	3	0	0	2	1	0	0	2	0	0	1	0	0	2	0	0	1	0	0	2	0	0	0	1	0	0	1	0	0	2	0	0	0	2	0	0	3	0	0	0	2
1	0	0	1	0	0	1	0	0	2	0	0	2	0	0	3	0	0	0	1	0	0	2	0	0	1	0	0	2	0	0	1	0	0	2	0	0	0	1	0	0	1	0	0	2	0	0	0	2	0	0	3	0	0	0	1
1	0	0	1	0	0	1	0	0	2	0	0	2	0	0	3	0	0	0	1	0	0	2	0	0	1	0	0	2	0	0	1	0	0	2	0	0	0	1	0	0	1	0	0	2	0	0	0	2	0	0	3	0	0	0	1
1	0	0	1	0	0	1	0	0	2	0	0	2	0	0	3	0	0	0	1	0	0	2	0	0	1	0	0	2	0	0	1	0	0	2	0	0	0	1	0	0	1	0	0	2	0	0	0	2	0	0	3	0	0	0	0
1	0	0	1	0	0	1	0	0	2	0	0	2	0	0	3	0	0	0	1	0	0	2	0	0	1	0	0	2	0	0	1	0	0	2	0	0	0	1	0	0	1	0	0	2	0	0	0	2	0	0	3	0	0	0	0
1	0	0	1	0	0	1	0	0	2	0	0	2	0	0	3	0	0	0	1	0	0	2	0	0	1	0	0	2	0	0	1	0	0	2	0	0	0	1	0	0	1	0	0	2	0	0	0	2	0	0	3	0	0	0	1
1	0	0	1	0	0	1	0	0	2	0	0	2	0	0	3	0	0	0	1	0	0	2	0	0	1	0	0	2	0	0	1	0	0	2	0	0	0	1	0	0	1	0	0	2	0	0	0	2	0	0	3	0	0	0	0
1	0	0	1	0	0	1	0	0	2	0	0	2	0	0	3	0	0	0	1	0	0	2	0	0	1	0	0	2	0	0	1	0	0	2	0	0	0	1	0	0	1	0	0	2	0	0	0	2	0	0	3	0	0	0	2
1	0	0	1	0	0	1	0	0	2	0	0	2	0	0	3	0	0	0	1	0	0	2	0	0	1	0	0	2	0	0	1	0	0	2	0	0	0	1	0	0	1	0	0	2	0	0	0	2	0	0	3	0	0	0	2
1	0	0	1	0	0	1	0	0	2	0	0	2	0	0	3	0	0	0	1	0	0	2	0	0	1	0	0	2	0	0	1	0	0	2	0	0	0	1	0	0	1	0	0	2	0	0	0	2	0	0	3	0	0	0	1
