# Supplementary material for: An end to the controversy over the microscopic detection and effects of pristine microplastics in fish organs
Source: Sci Rep. 2020 Jul 24;10:12434. doi: 10.1038/s41598-020-69062-3 (PMC7382502; doi:10.1038/s41598-020-69062-3)
Supplement: Supplementary file 1 — Supplementary Figures. [file 41598_2020_69062_MOESM1_ESM.docx]

***An end to the controversy over the microscopic detection and effects of pristine microplastics in fish organs.***

De Sales-Ribeiro, Carolina^1^, Brito Casillas, Yeray^2^, Fernandez, Antonio^1^, Caballero, María José^1^

^1^ Division of Veterinary Histology and Pathology, Institute for Animal Health and Food Safety (IUSA), Veterinary School, University of Las Palmas de Gran Canaria, Arucas, 35413, Spain

^2^ Research Institute in Biomedical and Health Sciences (IUIBS), University of Las Palmas de Gran Canaria, Las Palmas de Gran Canaria, 35016, Spain

**Supplementary Information**

A. **Supplementary Figures**

**Supplementary Figure S1.** Plastic materials by confocal fluorescence microscopy. (A) Green fluorescent microbeads, (B) polyethylene microfragments from a cosmetic body cleanser and (C) synthetic textile microfibers.

|  A  B  C |  |  |
| --- | --- | --- |

**Supplementary Figure S2.** Acute Experiment. (A) The percentage of fish with cosmetic and fluorescent microplastics (MP) in the different intestinal segments, at different hours post-feeding (hpf). (B) The percentage of fish with cosmetic and fluorescent MPs in the intestinal mucosa, at different hpf. (L. Propria: Lamina propria).

B

**Fish (%)**

**Intestinal segments, hours post-feeding (h)**

A

|  | Cosmetic MP | Fluorescent MP |
| --- | --- | --- |
| 2h Enterocytes | 0 | 66.66 |
| 2h Goblet Cells | 0 | 100 |
| 2h L. Propria | 0 | 0 |
| 6h Enterocytes | 0 | 66.6667 |
| 6h Goblet Cells | 0 | 100 |
| 6h L. Propria | 0 | 0 |
| 10h Enterocytes | 0 | 100 |
| 10h Goblet Cells | 0 | 100 |
| 10h L. Propria | 0 | 66.66 |
| 12h Enterocytes | 0 | 100 |
| 12h Goblet Cells | 0 | 100 |
| 12h L. Propria | 0 | 33.33 |
| 24h Enterocytes | 0 | 0 |
| 24h Goblet Cells | 0 | 0 |
| 24h L. Propria | 0 | 0 |

|  | Cosmetic MP | Fluorescent MP |
| --- | --- | --- |
| 2h Anterior | 0 | 33.3333 |
| 2h Middle | 75 | 66.6667 |
| 2h Posterior | 0 | 0 |
| 2h Rectum | 0 | 0 |
| 6h Anterior | 0 | 0 |
| 6h Middle | 100 | 66.6667 |
| 6h Posterior | 0 | 66.6667 |
| 6h Rectum | 0 | 0 |
| 10h Anterior | 0 | 0 |
| 10h Middle | 16.6667 | 0 |
| 10h Posterior | 100 | 100 |
| 10h Rectum | 0 | 66.6667 |
| 12h Anterior | 0 | 0 |
| 12h Middle | 0 | 0 |
| 12h Posterior | 100 | 0 |
| 12h Rectum | 0 | 66.6667 |
| 24h Anterior | 0 | 0 |
| 24h Middle | 25 | 0 |
| 24h Posterior | 75 | 0 |
| 24h Rectum | 0 | 0 |

**Fish (%)**

**Intestinal mucosa, hours post-feeding (h)**

**Supplementary Figure S3.** Acute Experiment. Panoramic sections of whole intestine by confocal microscopy. Fish fed fluorescent MPs (A) 6 hpf, fluorescent microspheres in the mid and posterior intestine (arrow), (B) 12 hpf, MPs in final portion of the posterior intestine (arrow) and (C) 24 hpf, MPs are absent from the intestine. Fish fed cosmetic MPs (D) 6 hpf, plastic microfragments in the mid and posterior intestine (arrow), (E) 12 hpf, MPs in the mid and posterior intestine (marked in red) and (F) few MPs remained in the intestinal lumen after 24 hours (arrow).

|  | | | **6 hpf** | **12 hpf** | **24 hpf** |
| --- | --- | --- | --- | --- | --- |
| **FLUORESCENT MP** |    A  B  C  D  E  F | | | | |
| **COSMETIC MP** | | * | | | |

**Supplementary Figure S4.** Acute Experiment. Confocal microscopy Z-stack images of Figure 1B. A fluorescent particle appears to have crossed the epithelial barrier (between 2.70 μm and 4.05 μm). A=0.45μm, B=0.90μm, C=1.35μm, D=1.80μm, E=2.25μm, F=2.70μm, G=3.15μm, H=3.60μm, I=4.05μm, J=4.50μm, K=4.95μm, L=5.40μm (Total z-stack = 8.55 μm, scaling=0.45μm).

A

E

J

F

K

G

L

H

D

C

B

I

**Supplementary Figure S5.** Acute Experiment. Confocal microscopy Z-stack images of Figure 1D. Fluorescent particles appeared to have cross the epithelial barrier, reaching the cytoplasm of the enterocytes and the lamina propria (between 1.35 μm and 2.25 μm). A=0.45μm, B=0.90μm, C=1.35μm, D=1.80μm, E=2.25μm, F=2.70μm, (Total z-stack = 10.8 μm, scaling=0.45μm).

A

E

F

D

C

B

**Supplementary Figure S6.** Acute Experiment. Confocal microscopy Z-stack images of Figure 1E. Fluorescent particles in the lamina propria (between 1.80 μm and 3.15 μm). A=0.90μm, B=1.35μm, C=1.80μm, D=2.25μm, E=2.70μm, F=3.15μm, G=3.60μm, H=4.05μm. (Total z-stack=9.9 μm, scaling=0.45μm).

A

E

F

G

H

D

C

B

**Supplementary Figure S7.** Acute experiment. Faces collected from fish fed fluorescent MPs under confocal microscope (A) 6 hpf, (B) 10 hpf, (C) 24 hpf, and (D) 48 hpf.

A

B

CV

D

Figure 5

**Supplementary Figure S8.** Acute experiment. Faces collected from fish fed cosmetic MPs and fibres under confocal microscope (A) 10 hpf and (B) 24 hpf.

B

A

A

**Supplementary Figure S9.** Sub-chronic experiment. Confocal microscopy Z-stack images of Figure 3A. Fluorescent particles in the liver (between 4.05 μm and 6.75 μm). A=3.15μm, B=3.60μm, C=4.05μm, D=4.50μm, E=4.95μm, F=5.40μm, G=5.85μm, H=6.30μm, I=6.75μm, J=7.20μm (Total z-stack=8.55 μm, scaling=0.45μm).

A

E

J

F

G

H

D

C

B

I

**Supplementary Figure S10.** Sub-chronic experiment. Intestine 15 days after depuration. (A) Cosmetic MPs (arrow) in the intestinal lumen, under a stereomicroscope. (B) Histological section of the intestine with cosmetic MP (arrow) (scale bar = 500 µm, H&E).

B

A

BV

B. **Supplementary Data**

**Supplementary Data S1.** FTIR spectrum of cosmetic plastic samples. Presence of dominant bands in the 3000, 2840, 1714 and 1470 cm^-1^. These characteristic bands present similarities with polyethylene (PE) polymers. <https://www.azom.com/article.aspx?ArticleID=12386>

**Supplementary Data S2.** FTIR spectrum of fluorescent plastic sample. Presence of dominant bands below 1600 cm^-1^ and weak bands in the 2922 and 3406 cm^-1^. The spectrum shows similarities with polyethylene glycol (PEG) polymers.

<https://www.researchgate.net/publication/274743255_PEGylated_magnetic_nanoparticles_PEGFe3O4_as_cost_effective_alternative_for_oxidative_cyanation_of_tertiary_amines_via_CH_activation>. Other common polymers were not detected.
